# Structural identifiability of constitutive relations from indirect field observations

**DOI:** 10.1093/pnasnexus/pgag281

**Published:** 2026-08-21

**Authors:** Y Sungtaek Ju

**Affiliations:** Department of Mechanical and Aerospace Engineering, University of California, Los Angeles, CA 90095, USA

**Keywords:** constitutive relations, Bayesian inverse problems, sparse Gaussian processes, optimal experimental design, PDE-constrained identification

## Abstract

Constitutive relations—functions linking a local state variable to a material or system property such as thermal conductivity, hydraulic permeability, or reaction rate constant—appear across the physical, engineering, and life sciences. They must often be inferred from sparse, indirect observations of the field they govern rather than from direct assays. Unlike the well-studied spatially varying parameter ϕ(x), the scalar state-dependent function $\phi (u)\;:\;R\to R^{+}$ of a single local state variable has received no systematic Bayesian treatment, and a basic question remains open: how much can sparse indirect observations actually inform ϕ(u)? We show the answer is governed by a two-factor attenuation: information must pass first through the inverse of the linearized partial differential equation (PDE) Jacobian and then through the sparse observation mask. The product of these factors lies far below unity for practical configurations, leaving the posterior structurally prior-dominated regardless of the number of indirect observations of the same type and configuration. The primary determinant is not the PDE class but the *coupling mechanism*—whether ϕ(u) enters the residual through a spatial gradient (as for diffusion coefficients) or pointwise (as for source terms). We instantiate the analysis in a sparse variational Gaussian process with an explicit, checkable prior and a closed-form Laplace posterior, and validate it across four PDE classes, four constitutive shapes, and two prior conditions. A small number of direct constitutive measurements at well-placed state values, bypassing the PDE entirely, collapse the posterior where arbitrarily many indirect observations of the same type cannot.

Significance statement Identifying how a material or system property depends on local state—how thermal conductivity varies with temperature, permeability with saturation, or reaction rate with concentration—is a routine challenge across physics, engineering, and life sciences. We treat a scalar property depending on a single local state variable. Current practice collects more field data and fits more flexible models. We show this is bounded by a structural limit: information about the unknown function is filtered twice before reaching the data, so no amount of additional field observation of the same type and configuration can close the gap. A handful of direct measurements at well-chosen state values bypasses the limit entirely, reframing experimental design for a broad class of inverse problems.

## Introduction

Many physical systems are governed by a *constitutive relation*—a scalar function ϕ(u) that maps a single local state variable *u* (temperature, pressure, concentration, and strain) to a material or system property such as thermal conductivity, hydraulic permeability, viscosity, or reaction rate constant. In practice this function is rarely available from direct assays. It must instead be inferred from sparse, indirect observations of the field it governs: a sensor array measuring temperature, a transducer network measuring pressure, a tracer experiment measuring concentration. The same underlying problem arises in thermal characterization of composite materials, permeability mapping in subsurface hydrology, growth-rate inference in tumor and ecological models, and viscosity identification in polymer processing. In each case a partial differential equation (PDE) F(u,ϕ(u))=0 must be satisfied, a sparse observation Cu≈y must be matched, and the unknown function $\phi \;:\;R\to R^{+}$ must be recovered. What distinguishes this problem from the well-studied identification of a spatially varying coefficient ϕ(x) is that *ϕ* is defined over the *range* of the field rather than over its spatial domain—constrained only at state values actually attained during the experiment—and *doubly indirect*: information about ϕ(u) must propagate through the PDE operator and then be filtered through the sparse observation mask before reaching the data.

A family of methods based on automatic differentiation now routinely returns point estimates of ϕ(u) jointly with the field, including physics-informed neural networks (1, 2) and the optimization-of-a-discrete-loss (ODIL) framework (3), but provides no uncertainty quantification. The deterministic uniqueness theory for ϕ(u) (4–7) establishes when recovery is possible in principle, and adjoint-based optimization frameworks (8, 9) demonstrate how to carry it out. Neither body of work, however, asks how much a realistic sparse indirect dataset *informs* the posterior, why that informativeness is structurally limited, or what type of measurement could resolve the limitation. The infinite-dimensional Bayesian inverse problem literature (10–12) addresses these questions for ϕ(x), but the ϕ(u) case demands different prior geometry (smoothness in function-value space, not position space), different identifiability theory (Cannon-type range restriction rather than Dirichlet-to-Neumann maps), and a different account of posterior informativeness.

The central result of this article is that the posterior for ϕ(u) is structurally prior-dominated by a two-factor attenuation. Information about ϕ(u) must first survive inversion through the linearized PDE Jacobian—a factor we denote $r_{1}$—and then survive the sparse observation mask—a factor $r_{2}$. Both factors are individually small, and their product is bounded well below unity for any practical configuration. As a result, the data Hessian cannot dominate the prior Hessian regardless of how many indirect observations of the same type are collected. The primary structural determinant of $r_{1}$ is not the PDE type (parabolic, elliptic, and hyperbolic) or the constitutive shape but the *coupling mechanism*: whether ϕ(u) enters the PDE residual through a spatial gradient—as for diffusion coefficients k(u)∇u—or pointwise—as for source terms f(u). Gradient coupling concentrates the parameter sensitivity on high-frequency modes that the PDE Jacobian most strongly attenuates; pointwise coupling distributes it more uniformly.

Three concrete tools are employed for this analysis. We parameterize ϕ(u) with a sparse variational Gaussian process (SVGP) whose mean function is a fixed analytical function, making the prior explicit, plottable, and directly checkable rather than implicit in a network architecture. We deliver the Laplace approximation to the posterior at the joint optimum analytically through the implicit function theorem (IFT), at no additional computational cost beyond the optimization itself. And we show that direct constitutive measurements—observations of $\phi (u^{*})$ at chosen state values $u^{*}$—contribute through the GP kernel alone, bypassing the PDE Jacobian entirely, so a small number of well-placed direct measurements collapses the posterior where indirect observations cannot.

We present the experimental evidence first and the theorem as its explanation. In Results, we report an experimental campaign across four PDE classes, four constitutive shapes, and two (+ two intermediate) prior conditions; introduce the structural attenuation theorem and its corollaries; report two tailored diagnostics (spectral concentration and Jacobian amplification) for identification difficulty and convergence failure; and develop the optimal experimental design (OED) framework for direct measurements. In Methods, we detail the architecture, joint optimization scheme, and benchmark construction. The Discussion positions the findings against existing work on ϕ(u) identifiability, Bayesian inverse problems, and PDE-constrained OED, and offers practical recommendations. Proofs and supporting heat-equation analysis are presented in the Supplementary material.

## Results

We applied the framework to four benchmark PDEs that span the standard operator classes—parabolic diffusion (heat equation, $u_{t}=\nabla \cdot (k(u)\nabla u)$); elliptic diffusion (Darcy, $-\nabla \cdot (k(u)\nabla u)=f_{\mathrm{src}}$); parabolic reaction–diffusion (Fisher–Kolmogorov, Petrovsky and Piskunov [KPP]), $u_{t}=Du_{xx}+f(u)$); and hyperbolic wave (Klein–Gordon, $u_{tt}=c^{2}u_{xx}+f(u)$)—and crossed each with four qualitatively distinct constitutive shapes: *baseline* (broad sensitivity), *concentrated* (narrow sensitivity), *bimodal* (two peaks), and *asymmetric* (skewed peak). Each combination was run under two prior conditions: a MISCAL (miscalibrated) prior with the correct topological structure but 50% amplitude error, and a FLAT (uninformed constant) prior with no shape information. Two intermediate priors are also examined. Across all 32 conditions we used the same SVGP with M=8 inducing points and $N_{\mathrm{obs}}=200$ randomly placed indirect observations; full architectural and benchmark details are given in Methods.

### Recovery patterns

**Figure 1:**
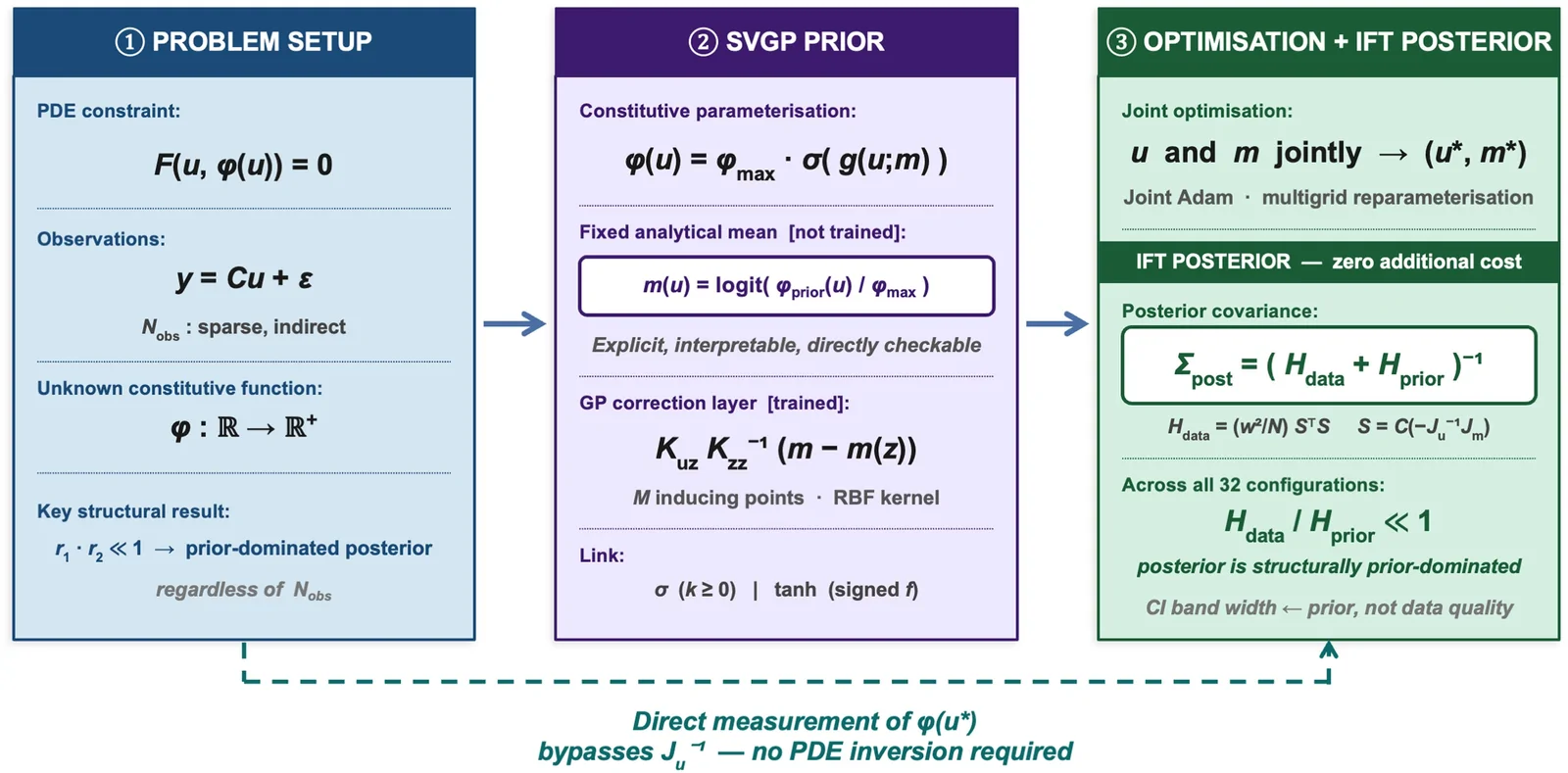
Framework overview. The SVGP–IFT inference pipeline. Left: the PDE F(u,ϕ(u))=0 is observed only through a sparse array *C* ($N_{\mathrm{obs}}=200$), so ϕ(u) is doubly indirect. Center: the SVGP prior $\phi (u)=\phi_{\max}\sigma (g(u;m))$ with a fixed analytical mean $m(u)=\mathrm{logit}(\phi_{\mathrm{prior}}(u)/\phi_{\max})$ and a trained GP correction. Right: joint optimization to $\left(u^{*},m^{*}\right)$ and the analytic IFT posterior $\Sigma_{\mathrm{post}}=(H_{\mathrm{data}}+H_{\mathrm{prior}}{)}^{-1}$. The dashed OED branch shows how direct measurements at $u^{*}$ bypass $J_{u}^{-1}$.

**Figure 2:**
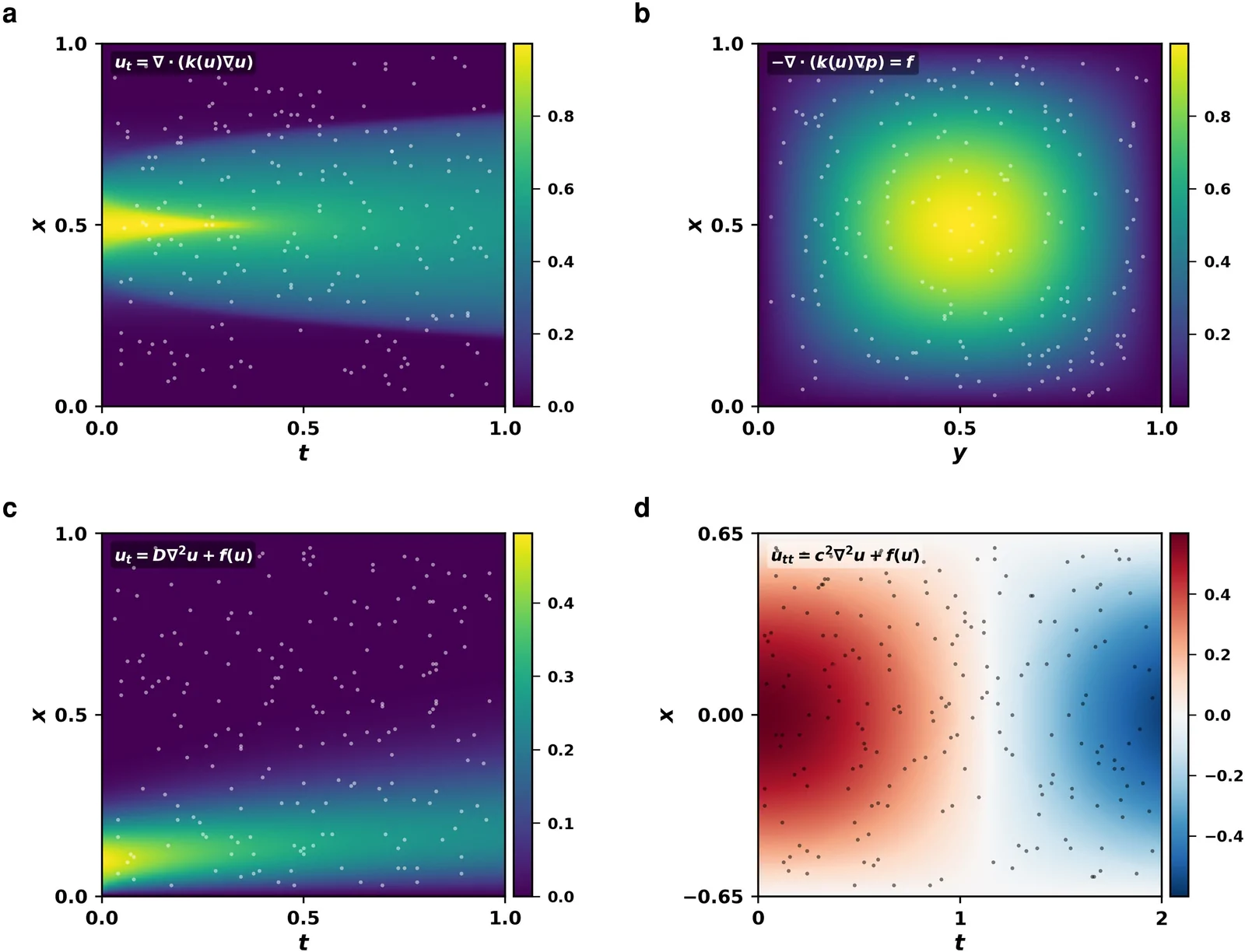
Ground-truth solution fields *u* for the four benchmark PDEs, with 200 randomly placed indirect observation locations (dots). All panels use the baseline constitutive function. a) Heat equation: space-time field u(x,t) on [0,1]×[0,1]; the Gaussian initial pulse decays and spreads, visiting u∈[0,0.55] with the highest values concentrated at t≈0. b) Darcy equation: steady-state spatial field u(x,y)=sin(πx)sin(πy) on $[0,1{]}^{2}$; the sinusoidal structure covers the full range u∈[0,1]. c) Fisher–KPP: reaction–diffusion front propagating rightward on [0,1]×[0,1]; logistic growth saturates near u≈0.35. d) Wave equation (Klein–Gordon): oscillatory space-time field u(x,t) on [0,1]×[0,2] with sign reversals (RdBu colorscale). The sparse observation dots relative to the rich 2D field structure illustrate the doubly indirect observation problem: constitutive information must propagate through both the PDE operator and the sparse observation mask before reaching the data.

Figure 1 summarizes the inference pipeline, and Fig. 2 shows the ground-truth solution fields for the four benchmarks alongside the 200 sparse observation locations.

Under the MISCAL prior the framework recovers ϕ(u) across all 32 conditions with the peak-normalized errors reported in Table 1. Errors range from 0.7% (Darcy bimodal) to 12.2% (wave bimodal), with most conditions below 5%. A miscalibrated prior that supplies the correct topological structure—monotonicity, the right number of peaks, the right qualitative skew—is therefore enough for the framework to refine the constitutive function from sparse indirect data, even when the prior amplitude is wrong by half.

**Table 1.** MISCAL recovery: *ϕ* RMSE (%), 5 seeds, 30,000 steps. All values peak-normalized.

Shape	Heat (*k*)	Darcy (*k*)	RD (*f*)	Wave (*f*)
Baseline	2.8±0.3	6.1±0.5	1.1±0.2	9.2±1.0
Concentrated	8.9±0.8	2.3±0.2	2.3±0.3	9.4±0.9
Bimodal	1.7±0.2	0.7±0.1	5.9±0.6	12.2±1.1
Asymmetric	2.5±0.3	2.7±0.3	1.3±0.2	5.3±0.5

Removing the topology—replacing the MISCAL prior with a FLAT constant prior that supplies no shape information at all—changes the picture dramatically and nonuniformly across PDE classes. Table 2 reports the ratio of FLAT to MISCAL RMSE for each condition, which we will call the *topology penalty*.

**Table 2.** FLAT/MISCAL topology penalty. Values >1 indicate that supplying the correct topology improves recovery; values <1 indicate the opposite. Bold: penalty ratio >20×.

Shape	Heat	Darcy	RD	Wave
Baseline	7.3×	6.0×	10.1×	1.6×
Concentrated	5.4×	11.8×	6.8×	2.4×
Bimodal	41.8×	74.1×	8.3×	0.9×
Asymmetric	14.9×	11.0×	10.7×	2.3×

Three observations stand out. First, all eight diffusion-coefficient conditions (heat and Darcy) show topology penalties of 5.4–74×: removing the topology destroys recovery. Second, the eight reaction–diffusion conditions show penalties of 6.8–10.7×, also consistent and substantial. Third, the eight wave-equation conditions show much milder penalties of 0.9–2.4×, and one wave condition—bimodal—is the lone case in the entire campaign where FLAT marginally outperforms MISCAL.

The most striking pattern is what we will call the *bimodal paradox*: bimodal ϕ(u) is the easiest of the four shapes to recover under a MISCAL prior for both diffusion-coefficient PDEs (heat 1.7%, Darcy 0.7%) but the *hardest* of the four shapes when topology is removed (heat 41.8×, Darcy 74.1× penalty ratios). The same structural feature—two well-separated peaks creating diverse sensitivity directions—that makes identification easy when the optimizer starts from the right qualitative shape makes it catastrophic when the optimizer must first discover the symmetry-breaking configuration from a flat start. The two-peak structure does not help an optimizer that has not yet decided there are two peaks.

A second pattern is the heat–Darcy concentrated reversal. The concentrated (sharp transition) shape is the hardest MISCAL condition for the heat equation (8.9%) but the second-easiest for Darcy (2.3%). The mechanism is coverage: the Darcy manufactured solution u=sin(πx)sin(πy) probes the full range u∈[0,1] throughout the domain, so the sigmoid transition is well-sampled everywhere simultaneously. The heat equation’s decaying Gaussian solution visits the transition region only transiently, concentrating sensitivity into a narrow spectral band that the PDE Jacobian inverse strongly attenuates. The same constitutive shape is easy or hard depending on how thoroughly the PDE solution samples its active range.

**Figure 3:**
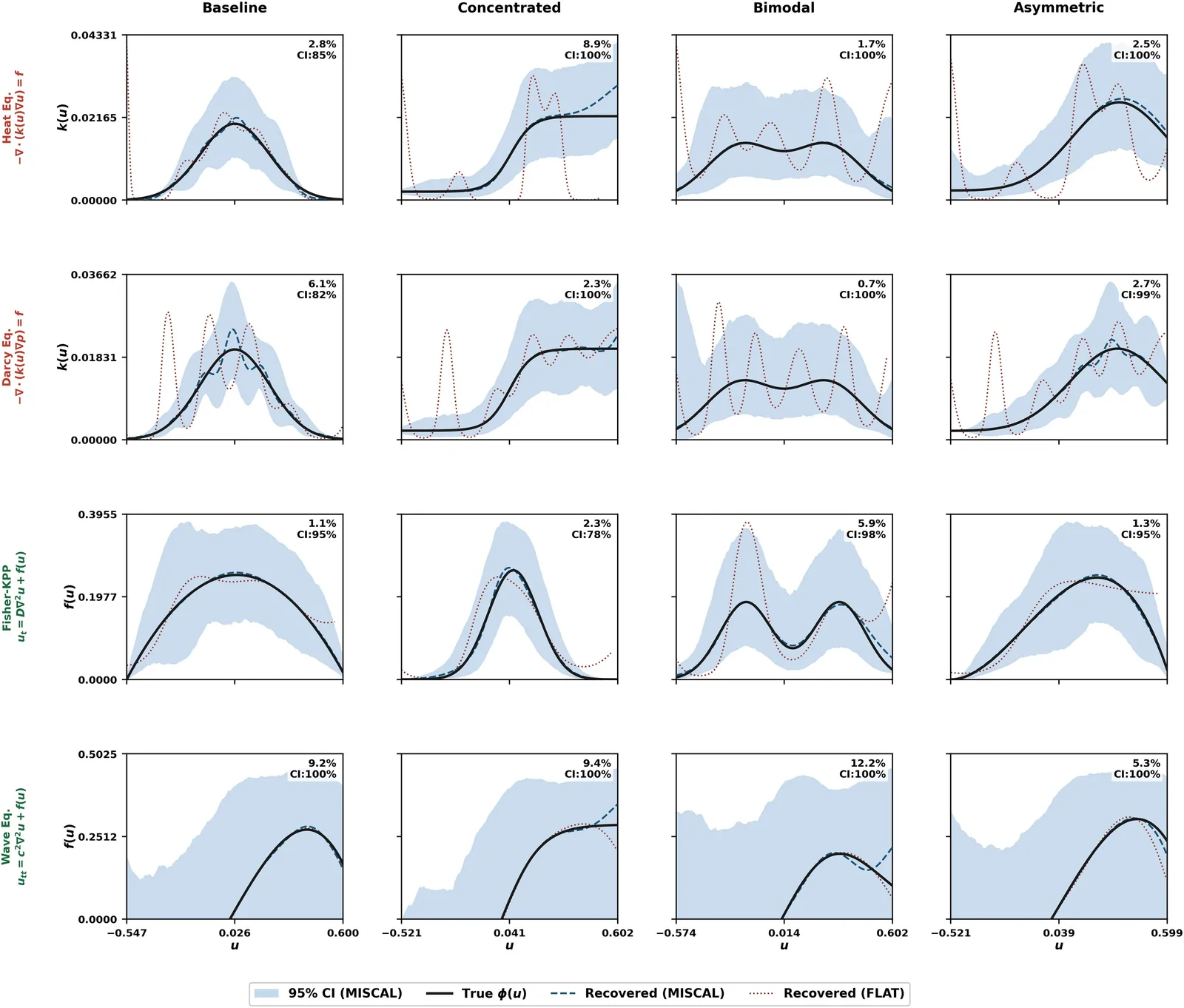
Recovery gallery under the MISCAL prior (5 seeds). Each panel: true ϕ(u) (black), MISCAL posterior mean (blue dashed), 95% IFT credible interval (blue shaded), and FLAT recovery (red dotted); *ϕ*-RMSE and CI coverage annotated. Rows: heat k(u), Darcy k(u), Fisher–KPP f(u), wave f(u). Columns: baseline, concentrated, bimodal, asymmetric. Coverages of 78–85% for some heat/Darcy baselines reflect Gauss–Newton overconfidence when $H_{\mathrm{data}}/H_{\mathrm{prior}}\ll 1$.

Figure 3 shows the full 16-panel MISCAL recovery gallery alongside the FLAT recoveries for comparison. The blue credible-interval bands are bounded by the nominal prior ($\sigma_{f}=0.5$, ℓ=0.10) and contain the truth in 78–100% of the MISCAL cases. The FLAT recoveries (red dotted) tell a consistent story: for the eight diffusion-coefficient configurations the recovered curves either stay flat or oscillate wildly, while for source-term PDEs some FLAT recoveries are nearly correct and others are poor.

**Figure 4:**
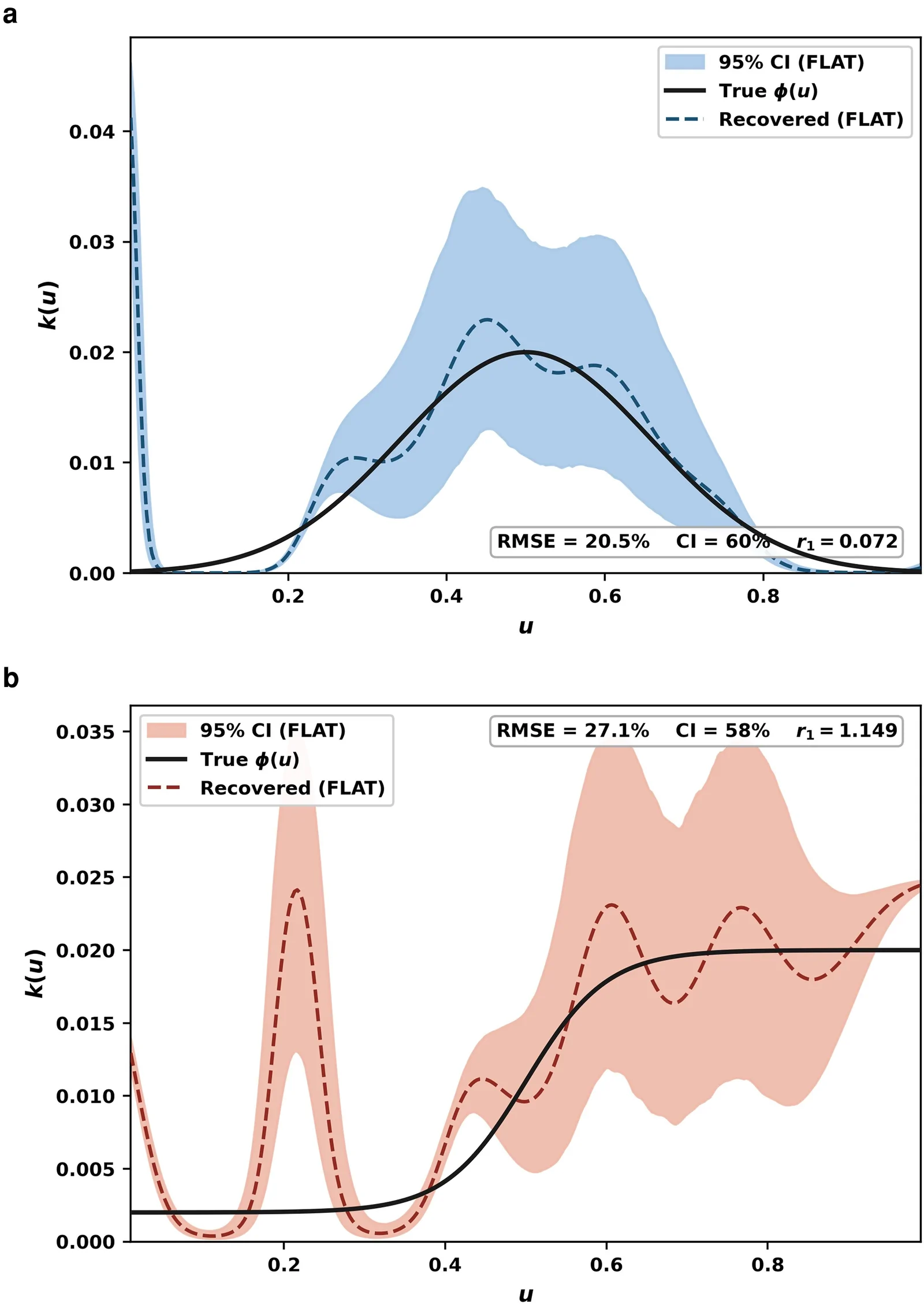
Two FLAT failure modes; both panels show true ϕ(u) (black), FLAT posterior mean (dashed), 95% IFT credible interval (shaded), and a stats box (RMSE, coverage, $r_{1}$). a) *Diffuse prior-dominated* (heat/Gaussian): a wide, honestly uninformative CI. b) *Overconfident false optimum* (Darcy/sigmoid): a comparably wide CI centered on a wrong recovered mean, with $r_{1}=1.149>1$ signaling Jacobian amplification. As detailed in the text, $r_{1}>1$ diagnoses a wrong optimum only in conjunction with large residuals; a near-correct prior can give $r_{1}>1$ at accurate recovery (Table S1).

The FLAT failures themselves split into two qualitatively different modes (Fig. 4). In the first mode, exemplified by the heat equation, the IFT credible interval is very wide—spanning most of $\left[0,k_{\max}\right]$—and the posterior is honestly uninformative. One sees that the prior is inadequate. In the second mode, exemplified by Darcy, the credible interval is of comparable width but is centered on a recovered curve that bears no resemblance to the truth: the optimizer has converged to a wrong local minimum and the posterior is overconfident about it. This second mode is the dangerous one: the credible interval gives no warning. We return to its computable diagnostic in A wrong-optimum diagnostic section.

Together, Tables 1 and 2 establish three findings that the following subsections explain: a topological prior with miscalibrated amplitude is essentially always sufficient; the topology penalty is heavy and uniform for diffusion coefficients but mild and inconsistent for the wave equation; and the bimodal shape exhibits a qualitative reversal between the two regimes. None of this is an artifact of the SVGP architecture or any particular optimization choice. It is a structural consequence of how constitutive information propagates through PDE-constrained inverse problems, as discussed in The structural explanation section.

#### Intermediate priors

The MISCAL and FLAT priors are two endpoints of a continuum, and a natural question is how recovery behaves between them. We probed two intermediate-prior families, holding the campaign otherwise fixed: a *wrong-width* prior with the correct topology and amplitude but a spatial extent 1.6× too broad, and a *partial-topology* prior that supplies only part of the true topological structure (a unimodal prior for a bimodal truth, or a symmetric prior for an asymmetric truth, area-matched; defined only where topology is well posed, and not for the wave benchmark’s signed source terms). Recovery interpolates smoothly between the FLAT and MISCAL endpoints, and the two families separate the two axes of prior information cleanly. Getting the width wrong costs little: the wrong-width prior recovers to near-MISCAL quality, often better because it carries the correct amplitude, since the GP correction absorbs a scale error easily. Getting the topology only partly right is the consequential case: for the gradient-coupled diffusion PDEs the partial-topology prior recovers most but not all of the FLAT→MISCAL gap (heat bimodal 11.9% against FLAT 71.1% and MISCAL 1.7%; Darcy bimodal 7.7% against 51.9% and 0.7%). Most tellingly, the coupling dichotomy established in The structural explanation section reappears within this experiment: for the pointwise-coupled reaction benchmark the partial-topology prior contributes little beyond FLAT, because the informative source-term data recover the missing structure without prior guidance. Full per-condition results are in Table S1.

### The structural explanation

All three findings—the universal sufficiency of topology, the heterogeneous topology penalty, and the bimodal paradox—share a common explanation. Information about ϕ(u) reaches the data along a single pathway: the parameter Jacobian $J_{m}=\partial F/\partial m$ generates a residual perturbation, which is mapped to a field perturbation by the inverse PDE Jacobian $J_{u}^{-1}$, which is then sampled by the sparse observation operator *C* to give the observed sensitivity matrix $S=C(-J_{u}^{-1}J_{m})$. The data Hessian of the IFT posterior is $H_{\mathrm{data}}=(w^{2}/N_{\mathrm{data}})\;S^{T}S$, and the prior Hessian is the inducing-point precision $H_{\mathrm{prior}}=K_{zz}^{-1}$. The ratio $H_{\mathrm{data}}/H_{\mathrm{prior}}$ determines whether the data or the prior controls the posterior.

Theorem 1 **(Two-factor structural attenuation).**

Let $J_{u},J_{m}$ be the field and parameter Jacobians at any converged joint optimum $\left(u^{*},m^{*}\right)$, and let $C\in \{0,1{\}}^{N_{\mathrm{obs}}\times N}$ select $N_{\mathrm{obs}}$ rows uniformly at random without replacement. Then, as an exact identity in expectation over the random observation mask, the expected squared Frobenius norm of the observed sensitivity matrix $S=C(-J_{u}^{-1}J_{m})$ factorizes as

$$E\left[\|S{\|}_{F}^{2}\right]=(r_{1}r_{2}{)}^{2}\;\|J_{m}{\|}_{F}^{2},$$

with

$$r_{1}\;:\;=\frac{\|J_{u}^{-1}J_{m}{\|}_{F}}{\|J_{m}{\|}_{F}},\;r_{2}\;:\;=\sqrt{\frac{N_{\mathrm{obs}}}{N}}.$$

The factor $r_{1}$ measures how much constitutive sensitivity survives inversion through the linearized PDE operator; $r_{2}$ measures how much of the surviving signal is captured by sparse observation. Their derivation is elementary—the $r_{2}$ part follows from uniform sampling, and the $r_{1}$ part is a definition—and the proof is in Section A1 (Observation 1). The *decomposition* above is exact; the accompanying claim that $r_{1}r_{2}\ll 1$ is an *empirical* property, established across our campaign and confirmed across the hyperparameter sweep (Table S2). A sharp analytic bound $r_{1}<1$ is proved only for the constant-coefficient Crank–Nicolson operator (Lemma S1); for the nonlinear case the Kirchhoff factorization (Section A2) gives structural insight but the resulting bound is loose, and the parabolic monotonicity underlying it remains a conjecture with empirical support (Section A5, see also Section A6). Both factors are individually small and bounded away from one for any practical configuration. For the heat-equation benchmark with the Gaussian baseline shape, the converged values are $r_{1}\approx 0.033$ and $r_{2}\approx 0.24$, giving a product of $r_{1}r_{2}\approx 0.008$ and a squared product of $∼6\times 10^{-5}$. The data Hessian is therefore at most a factor of $∼10^{-5}$ of the prior Hessian times $\|J_{m}{\|}_{F}^{2}/H_{\mathrm{prior},\max}$, regardless of how many indirect observations of the same type are added.

The decomposition is PDE-agnostic, but $r_{1}$ is not. It depends on the structure of $J_{m}$, which in turn depends on how ϕ(u) enters the PDE residual.

#### Coupling mechanism

For *gradient coupling*—*ϕ* entering through ∇⋅(k(u)∇u), as in the heat and Darcy equations—the parameter Jacobian carries the spatial gradient as a factor: $J_{m}\propto \nabla u\cdot \partial k/\partial m$. The gradient ∇u concentrates the energy of $J_{m}$ on the spatial modes where the solution varies most rapidly, which are precisely the high-frequency modes that the PDE Jacobian inverse most strongly attenuates—whether $J_{u}$ is the parabolic Crank–Nicolson operator (heat) or the elliptic symmetric positive definite (SPD) operator (Darcy). Heat and Darcy under MISCAL baseline conditions yield $r_{1}\approx 0.033\text{–}0.037$ and $H_{\mathrm{data}}/H_{\mathrm{prior}}∼10^{-5}\text{–}10^{-2}$.

For *pointwise coupling*—*ϕ* entering as a source term f(u), as in the Fisher–KPP and wave equations—$J_{m}$ involves only ∂f/∂m evaluated at the field. Without the ∇u factor, the energy of $J_{m}$ loads more uniformly across the spatial spectrum, and the lower-frequency components are not attenuated as strongly by $J_{u}^{-1}$. Reaction–diffusion and wave under MISCAL baseline conditions yield $r_{1}\approx 0.21\text{–}0.29$, an order of magnitude larger than the gradient-coupled case. Pointwise coupling does not eliminate the structural attenuation, but it reduces it by roughly an order of magnitude.

Table 3 reports the converged attenuation factors at the MISCAL baseline for all four PDEs.

**Table 3.** Attenuation factors across four PDE classes (MISCAL baseline shape, 5-seed mean).

Quantity	Heat	Darcy	RD	Wave
$r_{1}$ (Frobenius)	0.033	0.037	0.285	0.212
$r_{2}$ (empirical)	0.241	0.252	0.224	0.166
$r_{1}r_{2}$	0.008	0.009	0.064	0.035
$H_{\mathrm{data}}/H_{\mathrm{prior}}$ (empirical)	$1.78\times 10^{-6}$	$2.7\times 10^{-2}$	$3.0\times 10^{-6}$	$3.4\times 10^{-1}$

The data-to-prior ratio spans five orders of magnitude across PDEs, but no PDE achieves anything close to data dominance under the nominal prior. The wave equation sits at the top of the ratio not because $r_{1}$ is largest there but because its oscillatory dynamics cycle the field through the full constitutive range repeatedly, so $\|J_{m}{\|}_{F}$ accumulates contributions over time. Darcy sits second because its sinusoidal manufactured solution covers u∈[0,1] uniformly, also producing a large $\|J_{m}{\|}_{F}$. The reaction–diffusion equation is comparatively the most prior-dominated despite its moderate $r_{1}$, because its compact traveling front contacts the constitutive function only briefly and locally.

This is the explanation for the topology rule. For diffusion-coefficient PDEs the data carry an order of magnitude less information per observation than for source-term PDEs, so the prior must do an order of magnitude more work—including supplying the topology—for the posterior to converge. For wave-equation source terms the data carry enough information that even an uninformed FLAT prior can produce reasonable recovery from a partial topology discovery, which is why wave is the only PDE class with topology penalties below 3× across all shapes.

#### Two corollaries with practical consequences

Two corollaries of Theorem 1 sharpen the structural picture into actionable statements.

Corollary 1 **(Quantitative GP support restriction)**

Let $R=\mathrm{range}(u^{*})$ be the range of state values attained at convergence, and let $z_{j}$ be an inducing point with $d_{j}=\mathrm{dist}(z_{j},R)>0$. Then

$$|[J_{m}{]}_{(i,t),j}|\leq \phi_{\max}\|\sigma^{\prime }{\|}_{\infty }\|\nabla_{h}u^{*}{\|}_{L^{\infty }}\;e^{-d_{j}^{2}/(2\ell^{2})}\cdot \text{const.}$$

The IFT posterior is prior-dominated in the *j*-th GP mode, and no number of indirect field observations can provide information about ϕ(u) for u∉R.

Corollary 1 (proof in Section A1) is the quantitative Bayesian analog of the deterministic identifiability-interval restriction first established by Chavent and Lemonnier (6), who proved that the cost functional gradient vanishes identically for perturbations of *ϕ* supported outside the attained state range. Our result sharpens that qualitative vanishing into an explicit exponential decay rate governed by the GP kernel length scale and extends it from a property of the gradient to a property of the full Bayesian posterior. As a benchmark application: the heat equation visits R=[0.003,0.548], so inducing points $z_{7},z_{8}$ at u≈0.7,0.8 lie outside, with suppression factor $e^{-d_{7}^{2}/\ell^{2}}\approx 0.004$.

Corollary 2 **(Indirect-observation budget)**

The number of indirect observations required to bring $H_{\mathrm{data}}/H_{\mathrm{prior}}$ to unity scales as

$$N_{\mathrm{obs}}^{*}=\frac{NH_{\mathrm{prior},\max}}{r_{1}^{2}w^{2}\|J_{m}{\|}_{F}^{2}/N_{\mathrm{data}}}.$$

For the heat equation (Gaussian shape, MISCAL), $N_{\mathrm{obs}}^{*}\approx 10^{8}$.

Corollary 2 quantifies what Theorem 1 implies in operational terms. To overcome the structural attenuation by indirect observations alone—to drive the posterior from prior-dominated to data-dominated using only sparse field measurements—one would need on the order of $10^{8}$ measurements for the heat-equation benchmark, which is infeasible in most realistic experiments. This is the precise quantitative instance of Tarantola’s principle (12) that ill-conditioned forward operators cannot be compensated by larger datasets of the same type. It also distinguishes the regime studied here from the low-rank informed regime characterized by Bui-Thanh et al. (13), in which a few eigenvalues of $H_{\mathrm{data}}$ are large while the rest are small. In the regime of Theorem 1, *all* eigenvalues of $H_{\mathrm{data}}$ are small, because both $r_{1}$ and $r_{2}$ are bounded away from one. We emphasize that this limits *redundant* data, not all data: the obstruction is to adding more observations of the same type and configuration, which leave $r_{1}$ and $r_{2}$ unchanged. Observations that alter the sensitivity matrix $S=C(-J_{u}^{-1}J_{m})$—a different observation type, loading path, boundary condition, or region of state space probed—can and do help, and the extreme case, a direct constitutive measurement that bypasses $J_{u}^{-1}$ entirely, is the subject of the Optimal experimental design section.

#### A wrong-optimum diagnostic

The two-factor decomposition holds at any converged point, but the value of $r_{1}$ at a wrong local minimum can be qualitatively different from its value at the correct one. For all four Darcy shapes under the FLAT prior, the SVGP converges to a wrong local minimum at which $r_{1}$ exceeds 0.5 and for two shapes $r_{1}>1$, meaning that the linearized PDE Jacobian *amplifies* the parameter sensitivity rather than attenuating it: $\|J_{u}^{-1}J_{m}{\|}_{F}>\|J_{m}{\|}_{F}$. This pathology occurs because the FLAT-initialized elliptic operator at a wrong field configuration has near-zero eigenvalues, making $J_{u}^{-1}$ large. The signature in the IFT posterior is $H_{\mathrm{data}}/H_{\mathrm{prior}}>1$, which nominally indicates data dominance but here occurs together with high RMSE (27–52%)—a combination that reveals the false optimum. Such $r_{1}>1$ together with poor residuals should be interpreted as a wrong-local-minimum convergence, not as successful identification, and the appropriate remedy is a better prior initialization. The residual condition is essential: $r_{1}>1$ can also arise benignly when a near-correct prior yields a small parameter Jacobian $\|J_{m}{\|}_{F}$ (its denominator) while recovery remains accurate, as in the Darcy wrong-width case (Table S1). This is the diagnostic for the second failure mode of Fig. 4b.

The cross-condition $r_{1}$ values and their relationship to recovery quality are visualized in Fig. S1. The heatmap confirms the gradient/pointwise dichotomy at a glance and reveals the Darcy bimodal cell as the only gradient-coupling outlier with $r_{1}>0.5$, while the scatter shows that $r_{1}$ predicts RMSE within gradient coupling but not within pointwise coupling—foreshadowing the Spectral concentration section: the Frobenius $r_{1}$ is an aggregate, and the directional structure of the sensitivity matters separately.

### Spectral concentration

The Frobenius $r_{1}$ aggregates constitutive sensitivity across all GP directions. Two configurations with the same $r_{1}$ can have very different posteriors if one concentrates that sensitivity in a single direction and the other distributes it across many; $r_{1}$ across all 4×4 shape–PDE combinations is tabulated in Table S3.

Within each PDE, $r_{1}$ is correlated with RMSE quite differently depending on coupling type. For source-term PDEs (RD, wave) the within-PDE Spearman correlation is ρ≤−0.77: larger $r_{1}$ predicts smaller RMSE, as one would naively expect. For the heat equation ρ=+0.71: larger $r_{1}$ correlates with *larger* RMSE. The aggregate Frobenius factor is therefore not a universal predictor of recovery difficulty for diffusion coefficients.

A more discriminating diagnostic is the spectral concentration $\sigma_{1}^{2}(S_{\mathrm{obs}})/\sum_{j}\sigma_{j}^{2}(S_{\mathrm{obs}})$, which measures the fraction of the observed sensitivity carried by the dominant singular direction. It is tabulated across all 4×4 conditions in Table S4.

The most striking feature of the spectral-concentration data (Table S4) is the heat equation column. Three of the four PDEs show $\sigma_{1}^{2}/\Sigma$ in a narrow range of 0.74–1.00; the heat equation alone shows a factor-of-2.5 range from 0.39 to 0.98. Within heat, the low values are precisely the two shapes that break the spatial symmetry of the initial condition—concentrated sigmoid and asymmetric peak—while the symmetric shapes (Gaussian, bimodal) approach rank-1 with $\sigma_{1}^{2}/\Sigma \geq 0.93$. The mechanism is a symmetry-matching argument. The heat initial condition (IC) $u(x,0)=e^{-50(x-0.5{)}^{2}}$ is symmetric about x=1/2, so the field evolves symmetrically: u(x,t)=u(1−x,t) for all *t*. A symmetric constitutive function ϕ(u)≈ϕ(1−u) then makes inducing points $z_{j}$ and $z_{M+1-j}$ activate in lock-step at mirror-image spatial positions; their columns of $J_{m}$ are nearly parallel, and the sensitivity matrix is near-rank-1. An asymmetric constitutive function breaks this symmetry, and inducing points at different *u* values activate at genuinely different spatial positions, producing $J_{m}$ columns that point in independent directions and a distributed singular spectrum.

This mechanism is specific to time-dependent PDEs with symmetric initial conditions. The Darcy steady state observes all spatial points simultaneously, so its $\sigma_{1}^{2}/\Sigma$ stays in the high range regardless of constitutive symmetry. Pointwise coupling distributes sensitivity uniformly enough that the symmetry effect is invisible for RD and wave. The lone outlier is the RD concentrated case ($\sigma_{1}^{2}/\Sigma =0.997$): a sharp reaction rate activates only in a narrow *u*-band, so a single inducing point dominates regardless of symmetry—spectral concentration by domain restriction rather than by symmetry matching.

The practical implication runs counter to first intuition. A near-rank-1 sensitivity matrix means that all the constitutive information arrives along one direction in the GP space, where it is heavily attenuated by $r_{1}$. A distributed sensitivity spectrum means multiple GP directions carry independent information, and the posterior receives signal through several channels at once. *Low spectral concentration is favorable.* For diffusion-coefficient identification from a symmetric initial condition, asymmetric ϕ(u) produces multidirectional sensitivity that the scalar $r_{1}$ understates—and recovery is correspondingly easier than $r_{1}$ alone would predict.

### Optimal experimental design

Theorem 1 says that indirect observations cannot defeat the structural attenuation. Direct constitutive measurements—observations of $\phi (u^{*})$ at chosen state values $u^{*}$, with measurement noise *σ*—contribute through a qualitatively different channel. They add a rank-one term


$$H_{\mathrm{direct}}(u^{*},\sigma )=\frac{1}{\sigma^{2}}g(u^{*})g(u^{*}{)}^{T},\;g(u^{*})=\phi_{\max}\sigma^{\prime }(g(u^{*}))K_{zz}^{-1}K_{u^{*}z}^{T}\in R^{M},$$

to the posterior precision. The crucial feature is that $g(u^{*})$ depends only on the GP kernel: it bypasses $J_{u}^{-1}$ entirely. A direct measurement therefore avoids both attenuation factors $r_{1}$ and $r_{2}$ at once.

The *breakeven precision*  $\sigma^{*}$ is the value at which a single measurement at the D-optimal location turns a prior-dominated posterior into a data-dominated one:


$$\sigma^{*}=\frac{\left\|g(u^{*})\right\|}{\sqrt{H_{\mathrm{prior},\max}}}.$$

For the heat-equation benchmark (Gaussian shape, MISCAL), the optimum is at $u^{*}\approx 0.54$ and $\sigma^{*}\approx 0.022$. Any direct measurement with noise below 2% of $\phi_{\max}$ at the right state value flips the posterior away from prior dominance with one observation. Table S5 traces this transition explicitly: as *σ* decreases from $10^{-2}$ to $5\times 10^{-4}$, the data-to-prior ratio rises from 5× to ∼2000×, and the credible-interval width drops by an order of magnitude. The right-hand column reports the equivalent number of indirect observations that would be needed to achieve the same posterior precision through Corollary 2—and that number runs from ∼200,000 to ∼78 million. One sufficiently precise direct measurement is worth millions of indirect ones.

For multiple direct measurements, the greedy D-optimal placement reduces to sequential rank-one updates of $\log\;\text{det}H_{\mathrm{post}}$, which can be evaluated in $O(nM^{3})$ time via the matrix determinant lemma. Table S6 reports the resulting placement sequence for the heat equation benchmark.

The sequence has three structural features. The algorithm tiles outward from the prior mass centroid at u≈0.54, placing each new measurement near the boundary of the previously informed region. Information saturates sharply at n=M=8 inducing points: steps 9 and 10 contribute 2.9% of the total information gain. And uniform placement over [0.1,0.9] achieves 97% of the greedy gain—the information landscape is smooth enough that any reasonable distribution of *M* measurements is near-optimal. The A-optimal trace falls from 193.6 at n=0 to 137.4 at n=1, 69.5 at n=5, and 0.67 at n=10. Ten well-placed direct measurements collapse the posterior to a near-deterministic point estimate.

The saturation and placement geometry are visualized in Fig. S2, and the cross-PDE benefit factors (from the same analysis across all four PDEs and shapes) in Table S7.

The wave equation benefits the most from direct measurements (3.3–8.9×), while heat, Darcy, and RD show more moderate but uniformly positive benefit (1.5–2.8×). The wave advantage is not because wave is the most prior-dominated—it is in fact the least, by Table 3—but because $g(u^{*})\propto \phi_{\max}$  $\sigma^{\prime }(g(u^{*}))K_{zz}^{-1}K_{u^{*}z}^{T}$, and the wave benchmarks use $\phi_{\max}=0.50$ versus 0.05 for heat and Darcy. A factor-of-ten larger $\phi_{\max}$ yields a factor-of-hundred larger $H_{\mathrm{direct}}$ relative to the same $H_{\mathrm{prior}}$, so each direct wave measurement carries proportionally more information. Bimodal and baseline shapes yield the most uniform benefit because their broad sensitivity is well matched to five uniform measurement locations; concentrated shapes yield the smallest benefit because the fixed smooth mean already captures the narrow transition.

## Methods

This section details the SVGP parameterization, the joint optimization scheme, the benchmark experimental design, and the OED formulation.

### Problem formulation and notation

Let Ω=[0,1], T>0, and let $F\;:\;R^{N}\times R^{M}\to R^{N}$ be the discretization of the governing PDE on an $N_{x}\times N_{t}$ (or $N_{x}\times N_{y}$) grid, with $N=N_{x}N_{t}$ (or $N_{x}N_{y}$). Throughout, ϕ(⋅) denotes the constitutive function (written k(⋅) for diffusion coefficients and f(⋅) for reaction or source terms); *u* is the state field; $m\in R^{M}$ is the GP variational mean; $J_{u}=\partial F/\partial u$ and $J_{m}=\partial F/\partial m$ are the field and parameter Jacobians; $C\in \{0,1{\}}^{N_{\mathrm{obs}}\times N}$ is the sparse observation operator; $S=C(-J_{u}^{-1}J_{m})$ is the observed IFT sensitivity matrix; and $H_{\mathrm{data}}$, $H_{\mathrm{prior}}$ are the data and prior Hessians of the IFT posterior. At the joint optimum $\left(u^{*},m^{*}\right)$ of the ODIL loss (3), the Laplace-approximated IFT posterior covariance is $\Sigma_{\mathrm{post}}=(H_{\mathrm{data}}+H_{\mathrm{prior}}{)}^{-1}$ with $H_{\mathrm{data}}=(w^{2}/N_{\mathrm{data}})\;S^{T}S$ and $H_{\mathrm{prior}}=K_{zz}^{-1}$.

### SVGP parameterization of the constitutive function

We parameterize the unknown constitutive function as a sparse variational Gaussian process (14) with a fixed analytical mean function:


$$\phi (u;m)=\phi_{\max}\sigma \left(g(u;m)\right),\;g(u;m)=m(u)+K_{uz}K_{zz}^{-1}\left(m-m(z)\right),$$

where *σ* is the logistic sigmoid (or tanh for signed source terms in the wave benchmark); $\phi_{\max}$ is a loose upper bound on *ϕ*; $z\in R^{M}$ are *M* inducing points; $m\in R^{M}$ is the variational mean of the GP at the inducing points (the only trainable GP parameter in our setup); and $K_{uz},K_{zz}$ are radial basis function (RBF) kernel matrices with length scale ℓ=0.10 and output scale $\sigma_{f}=0.50$. The fixed analytical mean function m(u) encodes the prior in logit space and is nontrainable. The architecture satisfies three requirements simultaneously: the prior is explicit and directly checkable, since m(u) is a smooth analytical function we plot and inspect; the IFT posterior is an M×M Laplace approximation, tractable precisely because *M* is small; and the sigmoid (or tanh) link enforces physical constraints (positivity or boundedness) without constrained optimization.

For the MISCAL prior, $m(u)=\mathrm{logit}(\phi_{\mathrm{miscal}}(u)/\phi_{\max})$, where $\phi_{\mathrm{miscal}}$ is a smooth function with the correct topological structure but deliberately miscalibrated amplitude—for the heat Gaussian shape, $\phi_{\mathrm{miscal}}(u)=0.03\;e^{-20(u-0.5{)}^{2}}$ (a 50% amplitude overestimate of $\phi_{\mathrm{true}}$). For the FLAT (uninformed) prior, m(u)≡0, so $\phi (u)=\phi_{\max}/2$ in the absence of GP correction. Because the mean function is nontrainable, the GP correction $K_{uz}K_{zz}^{-1}(m-m(z))$ starts near zero and remains small throughout training: the variational means m need only encode the residual between the prior and the truth, not the full constitutive shape.

We place M=8 inducing points uniformly at $z_{j}=0.10+$  0.80(j−1)/7, giving spacing δ/ℓ=1.14>1 and a well-conditioned kernel matrix ($\kappa (K_{zz})\approx 15$). Smaller *δ* produces near-rank-1 $K_{zz}$ and a pathological $H_{\mathrm{prior}}$; the resulting degradation is demonstrated by the hyperparameter sweep (Section S2).

#### Hyperparameter sensitivity

To confirm that the structural conclusion does not depend on the particular kernel length scale and inducing-point count, we swept ℓ∈{0.05,0.10,0.20} against M∈{6,8,12} on the heat and reaction–diffusion baselines under the MISCAL prior (Table S2). The attenuation is robust throughout: $r_{1}$ remains small (0.03–0.08 for heat, 0.17–0.37 for reaction–diffusion) and $H_{\mathrm{data}}/H_{\mathrm{prior}}$ stays far below unity ($10^{-3}$ to $10^{-8}$) in every cell, so prior-dominance is not an artifact of the nominal (ℓ,M). Point-estimate accuracy does depend on the pair through the conditioning of $K_{zz}$: increasing *M* at fixed domain shrinks the inducing-point spacing *δ* relative to ℓ, and once δ/ℓ≲1 the kernel matrix becomes near-rank-deficient and recovery degrades (heat, ℓ=0.05, M=12: RMSE 7.8%, coverage 79%). Larger ℓ tolerates larger *M*, and the credible-interval coverage tracks the degradation, correctly flagging the ill-conditioned cells. The operating recommendation is therefore to keep δ/ℓ≳1, which the campaign setting (M=8, ℓ=0.10) satisfies. In this study $H_{\mathrm{prior}}=K_{zz}^{-1}$ is evaluated at the same ℓ being swept, so that $H_{\mathrm{data}}/H_{\mathrm{prior}}$ is internally consistent across cells.

The combination of M=8 at δ≈ℓ restricts the GP posterior to constitutive functions whose variation scale exceeds approximately ℓ. Finer-scale structure cannot be represented and will be absorbed into the fixed mean or missed entirely; this is a model-class limitation distinct from the structural attenuation analyzed in the Results, and the recovered *ϕ* should be interpreted as an effective, model-consistent description rather than an intrinsic material property independent of the parameterization (15).

### Joint optimization

We minimize


$$L(u,m)=\|F[u,\phi (\cdot ;m)]{\|}^{2}+w^{2}\frac{\|Cu-\tilde{y}{\|}^{2}}{N_{\mathrm{data}}}+\lambda_{\mathrm{KL}}\;\mathrm{KL}(q\|p),$$

where w=2, $\tilde{y}$ are the noisy observations, and KL(q‖p) is the SVGP KL divergence evaluated analytically. The KL term is set at $\lambda_{\mathrm{KL}}=10^{-4}$. An ablation at $\lambda_{\mathrm{KL}}=0$ over all four heat-equation shapes (FLAT prior) shows that point-estimate accuracy is essentially unchanged (within ±8% of baseline), but the IFT posterior becomes systematically overconfident: CI coverage drops from ≈100% to 40–60%. The KL term plays a posterior-calibration role, not a regularization role; we retain it at $10^{-4}$ for meaningful uncertainty quantification (UQ).

Joint optimization of the field *u* and GP variational means m is essential. Alternating between inner and outer steps causes gradient decoupling—the field fully adapts to the current *ϕ* before *ϕ* is updated—which suppresses the gradient signal needed to improve the constitutive estimate, catastrophically so for uninformed priors. We therefore use joint Adam with two extensions. First, we reparameterize the field using a 6-level multigrid (3, 16): $u=\ell_{0}+\mathrm{upsample}(\ell_{1})+\cdots +{\mathrm{upsample}}^{5}(\ell_{5})$, where each $\ell_{k}$ is a coarse-grid array upsampled to full resolution by bilinear interpolation. The multigrid operates over space-time for the heat, RD, and wave benchmarks and over the 2D spatial domain for Darcy; the implementation is identical (a sum of jax.image.resize calls). Second, we use per-group cosine decay and gradient clipping: field parameters at ${\mathrm{lr}}_{f}=5\times 10^{-4}$, GP parameters at ${\mathrm{lr}}_{g}=2\times 10^{-3}$, both with gradient clipping at norm 2.0 and cosine decay to 1% of the initial learning rate over 20,000 steps. For the heat, RD, and wave benchmarks we warm-start the field from a forward solve using the prior mean. The Darcy prior diffusion coefficient is near zero at the Dirichlet boundaries, making any forward solve with a nondegenerate prior ill-conditioned, so we warm-start instead from the manufactured solution $u_{\mathrm{ref}}=\sin\;(\pi x)\sin\;(\pi y)$.

### Benchmark PDEs and experimental design

To test the generality of the framework, we ran the full pipeline across four PDE classes and four constitutive shapes per PDE, under both MISCAL (correct topology, 50% amplitude overestimate) and FLAT (constant, no shape information) priors: 5 seeds × 30,000 steps per condition, 32 conditions total. The four PDE classes are parabolic diffusion (heat equation, $u_{t}=\nabla \cdot (k(u)\nabla u)$); elliptic diffusion (Darcy, $-\nabla \cdot (k(u)\nabla u)=f_{\mathrm{src}}$); parabolic reaction–diffusion (Fisher–KPP, $u_{t}=Du_{xx}+f(u)$); and hyperbolic wave (Klein–Gordon, $u_{tt}=c^{2}u_{xx}+f(u)$). The four shapes per PDE are baseline (broad derivative support); concentrated (sigmoid for *k*, sharp Gaussian for RD *f*, softstep tanh for wave *f*); bimodal (two sensitivity peaks); and asymmetric (skewed peak). All *ϕ*-RMSE values are normalized by the peak of $\phi_{\mathrm{true}}$ over the active *u*-range. By design, the benchmarks use noiseless observations (noise enters only through observation sparsity) and synthetic ground truth with no model mismatch, so that the structural attenuation can be isolated and the factors $r_{1}$ and $r_{2}$ measured cleanly; the interaction of the attenuation with additive noise, model error, and experimental data is a subject of future study as mentioned in the Discussion section. PDE-specific parameters appear in Table 4. Shared SVGP hyperparameters across all conditions: M=8, ℓ=0.10, $\sigma_{f}=0.50$, $\lambda_{\mathrm{KL}}=10^{-4}$, w=2, $N_{\mathrm{obs}}=200$.

**Table 4.** Benchmark PDE parameters for the four experimental conditions. All conditions use M=8 inducing points and $N_{\mathrm{obs}}=200$ indirect observations. The coupling type determines the $r_{1}$ regime; $\phi_{\max}$ sets the constitutive output scale.

PDE	Coupling	Grid	*N*	$\phi_{max}$	Link
Heat (parabolic)	Gradient	64×64	4,096	0.05	Sigmoid
Darcy (elliptic)	Gradient	64×64	4,096	0.05	Sigmoid
Fisher–KPP (parabolic RD)	Pointwise	64×64	4,096	0.50	Sigmoid
Wave (Klein–Gordon)	Pointwise	64×128	8,192	0.50	Tanh

### Direct measurement OED: formulation

A direct constitutive measurement at state value $u^{*}$ with noise *σ* adds a rank-one term to the posterior precision:


$$H_{\mathrm{direct}}(u^{*},\sigma )=\frac{1}{\sigma^{2}}g(u^{*})g(u^{*}{)}^{T},\;g(u^{*})=\phi_{\max}\sigma^{\prime }(g(u^{*}))K_{zz}^{-1}K_{u^{*}z}^{T}\in R^{M}.$$

The gradient $g(u^{*})$ depends only on the GP kernel: it bypasses $J_{u}^{-1}$ entirely. The breakeven precision $\sigma^{*}=\|g(u^{*})\|/\sqrt{H_{\mathrm{prior},\max}}$ is the value at which a single direct measurement at the D-optimal location turns the posterior from prior-dominated to data-dominated—formally the pointwise inverse of Theorem 1 for direct observations. The full posterior precision becomes $H_{\mathrm{post}}=H_{\mathrm{data}}+H_{\mathrm{prior}}+\sum_{j}H_{\mathrm{direct}}(u_{j}^{*},\sigma )$, and the greedy D-optimal sequence is computed in $O(nM^{3})$ time via the matrix determinant lemma:


$$\log\;\text{det}\;H_{\mathrm{post}}(u_{1}^{*},\ldots ,u_{n}^{*})=\log\;\text{det}\;H_{0}+\sum_{\;j=1}^{n}\log\;\left(1+\frac{g(u_{j}^{*}{)}^{T}H_{\;j-1}^{-1}g(u_{j}^{*})}{\sigma^{2}}\right).$$

All OED computations are performed analytically postconvergence, using the converged $\left(u^{*},m^{*}\right)$ from the training runs.

## Discussion

The Results establish three things: that a topological prior with miscalibrated amplitude is essentially always sufficient for recovery; that the topology penalty separates by coupling mechanism rather than by PDE class; and that direct constitutive measurements at well-placed state values bypass the structural attenuation entirely. The Discussion takes these findings and asks what they mean for practice, where they sit relative to the existing literature, and what they leave open.

### Practical implications

The most consequential structural finding is that the coupling mechanism—whether ϕ(u) enters through ∇u or pointwise—dominates over the PDE type in determining identifiability. This has three practical implications worth stating explicitly, because they do not follow automatically from the existing literature.

The first is a planning principle for any new ϕ(u) identification problem. Before designing the experiment or choosing the inference method, examine the coupling: does the unknown enter the residual through a gradient or pointwise? For gradient coupling the data are an order of magnitude less informative per observation, the topological prior is essential and must be specified honestly, and the recovery strategy should budget for direct constitutive measurements rather than relying on field data alone. For pointwise coupling the data carry more information, partial topology discovery from uninformed initialization is possible, and indirect-only inference is sometimes adequate. The PDE class matters less than the term structure: heat and Darcy belong to the same identifiability regime despite being parabolic and elliptic, whereas Fisher–KPP and the Klein–Gordon wave equation share another regime despite being parabolic and hyperbolic.

The second implication is that uncertainty quantification cannot be omitted in the gradient-coupled regime. A point-estimate framework that returns no posterior cannot distinguish between a successful identification and a wrong-local-minimum convergence with high residuals, and the Darcy FLAT amplification cases show that the difference can be invisible to inspection of the recovered function alone. The IFT posterior is essentially free given the joint optimization machinery the PDE-discovery community already uses, and it provides a check that point estimates cannot.

The third implication is methodological pluralism. Direct constitutive measurements—pilot experiments, calibration assays, materials database queries—carry orders of magnitude more posterior information per observation than indirect field measurements, and they bypass the structural attenuation entirely. The natural workflow combines a small number of direct measurements (M=8 for our parameterization) with a larger number of indirect ones, rather than treating the two as substitutes. The OED framework translates this principle into a budget calculation.

#### What is structural and what is a modeling choice

It is worth separating the parts of our result that are structural from the parts that reflect modeling choices, since the distinction governs how far the conclusions travel. The two-factor attenuation $r_{1}r_{2}\ll 1$ is a structural property of the information pathway: $S=C(-J_{u}^{-1}J_{m})$ is fixed by the linearized operator and the observation geometry, and the decomposition of Theorem 1 holds for any parameterization. What is *not* purely structural are the specific posterior-dominance magnitudes, the credible-interval widths, and the recovered shapes, which additionally reflect the RBF kernel, the Laplace approximation, and the synthetic benchmark design. The hyperparameter sweep supports exactly this separation: across ℓ and *M* the structural quantities $r_{1}$ and $H_{\mathrm{data}}/H_{\mathrm{prior}}$ stay in the attenuated regime, while the accuracy figures move. Read this way, the article makes a strong structural claim about *whether* indirect field data can dominate the prior, and a more contingent, model-dependent claim about *how well* any particular recovery will do.

#### Mixed coupling

Our four benchmarks are each purely gradient- or purely pointwise-coupled, but many physical problems contain both mechanisms. The framework makes a concrete prediction for that case. When *ϕ* enters through both a gradient term and a pointwise term, the parameter Jacobian $J_{m}$ is a sum of a gradient-weighted contribution—concentrated on the high-frequency modes that $J_{u}^{-1}$ strongly attenuates—and a pointwise contribution that loads more uniformly across the spectrum. Because $r_{1}$ is a norm ratio dominated by the larger-norm contribution, the less-attenuated (pointwise) term tends to set the identifiability regime whenever its sensitivity is comparable in magnitude: a mixed-coupling problem should inherit the more favorable pointwise behavior to the extent the source-term sensitivity is non-negligible, and degrade toward the gradient-coupled regime as that contribution vanishes. We present this as a testable prediction of the taxonomy rather than a validated result; a controlled mixed-coupling benchmark is a natural follow-up.

#### When direct measurements are unavailable

The OED framework recommends direct constitutive measurements, but in some applications—the stress–strain law in solid mechanics is a natural example—truly direct assays are difficult or impossible. Our analysis does not imply such problems are unsolvable, but it does sharpen what solving them requires. Three consequences follow. First, the posterior remains prior-dominated, so the recovered *ϕ* is only as trustworthy as the prior topology; the practitioner must supply and defend a topological prior and must report the IFT posterior, which signals through wide or mis-centered credible intervals when that prior is inadequate. Second, the design lever does not disappear—it shifts from “direct versus indirect” to changing the indirect observation configuration so as to alter *S* itself (different loading paths, boundary conditions, or observation types that raise $r_{1}$), which is the informative-data principle formalized in the Results (Corollary 2 and the discussion following it). Third, for a genuinely gradient-coupled quantity observed only indirectly under a poor prior, the honest report is that the problem is structurally prior-dominated, rather than a confident point estimate that the credible interval cannot support. The absence of direct measurements therefore raises the premium on honest uncertainty quantification and a well-specified prior; it does not by itself render the problem intractable.

### Relation to prior work

Our result sits at the intersection of four bodies of work; we summarize the connections here and give the full discussion in Section B1. The classical deterministic theory of ϕ(u) identification (4–7, 17–19) establishes in-principle recoverability and the attained-range support restriction; two of its results have direct Bayesian analogs here (Chavent–Lemonnier’s gradient insensitivity becomes Corollary 1, and their level-set gradient representation is the continuous analog of our sensitivity matrix *S*). The adjoint-based reconstruction frameworks of Bukshtynov, Protas and collaborators and the all-at-once ODIL/PINN paradigm (1–3, 8, 9, 16, 20–25) supply the point-estimate machinery our Bayesian treatment extends; the structural attenuation applies to the entire class rather than to any one method. Within Bayesian inverse-problem theory (10–13), $H_{\mathrm{data}}/H_{\mathrm{prior}}$ is Tarantola’s data-resolution fraction, and our all-eigenvalues-small regime complements the low-rank informed regime of Bui-Thanh et al. Finally, our OED optimizes the observation *type* in the function-value domain rather than sensor placement in space (26–30), and connects to the nonexistence results of Matharu and Protas for turbulent-flow closures (31, 32).

### Recommendations

(i) Identify the coupling mechanism first: whether *ϕ* enters through ∇u or pointwise sets the regime; gradient coupling demands a topological prior and UQ-bearing inference, pointwise coupling is more forgiving. (ii) For diffusion coefficients, topology is non-negotiable: even a rough unimodal-vs-bimodal prior far outperforms a constant, with topology penalties exceeding 40× for bimodal heat/Darcy. (iii) For source terms, the IFT posterior stays calibrated even when the prior is wrong: all 16 source-term FLAT configurations yield ≥90% coverage, signaling prior inadequacy through wide bands. (iv) Budget about *M* direct measurements: information saturates at the number of inducing points (steps beyond contribute <3%), and uniform placement recovers 97% of the greedy gain for symmetric shapes. (v) Read $r_{1}$, $H_{\mathrm{data}}/H_{\mathrm{prior}}$, and the residual jointly: small $r_{1}$ with tiny ratio and a wide honest CI means structural prior-domination, whereas $r_{1}>1$ with large residuals means a wrong local minimum—but $r_{1}>1$ alone is not a failure signature, since a near-correct prior can give $r_{1}>1$ at accurate recovery (Table S1).

### Limitations and future directions

#### Architectural commitments

The framework commits in advance to a fixed analytical mean m(u), a kernel family (RBF), and fixed hyperparameters $\left(\ell ,\sigma_{f}\right)$. These are explicit and checkable by design, but the RBF kernel favors smooth shapes and the M=8 inducing points restrict the GP correction to variation scales above ℓ, so sharper true functions are absorbed into the mean or missed; the recovered *ϕ* should be read as the best approximation within the model class. Adaptive hyperparameters and richer kernels are natural extensions.

#### The Laplace approximation

The IFT posterior is a Gauss–Newton approximation. For strongly nonlinear constitutive functions or genuinely multimodal posteriors it may miss non-Gaussian features. CI coverage of 78–85% for some MISCAL configurations (heat and Darcy baselines) reflects this known overconfidence rather than a model failure—and is itself a diagnostic that the underlying problem is strongly prior-dominated.

#### Grid resolution, observation noise, and model mismatch

All experiments use moderate spatial grids, noiseless observations (noise enters only through observation sparsity), and synthetic ground truth with no model mismatch—a design choice that isolates the structural attenuation. Three settings therefore remain open. Additive observation noise: we expect it to *deepen* prior-dominance by further shrinking the effective data Hessian, but its interaction with the spectral attenuation is uncharacterized. Model mismatch: under a misspecified forward model the recovered *ϕ* should be read as an effective, model-consistent description rather than an intrinsic property. Experimental data, where noise and model error act together, is the most important practical test. The behavior of the spectral attenuation at higher grid resolutions is likewise open.

#### Scope and other open directions

The present analysis is confined to a scalar constitutive function of a single state variable. Multiargument constitutive relations $\phi (u_{1},u_{2})$—arising in coupled thermo-mechanical or multispecies reactive transport—and tensorial or history-dependent (rate- or path-dependent) laws are outside the present scope; whether the coupling-mechanism taxonomy generalizes to them is an open question that the multiargument case would begin to test. Application to experimental data, where model error and observation noise interact with the structural attenuation, is the most important practical test. The 32-experiment campaign reported here, with consistent definitions, controlled experimental design, and publicly specified constitutive shapes, is intended as a step toward the standardized benchmarks the state-dependent inverse problem community currently lacks (15, 33).

## Conclusions

The state-dependent constitutive identification problem ϕ(u) is structurally harder than its spatially varying counterpart ϕ(x), in a way that becomes precise once posed in Bayesian terms. The data Hessian for ϕ(u) is filtered by two factors in series: a PDE spectral factor $r_{1}$ that measures how much constitutive sensitivity survives inversion through the linearized PDE Jacobian, and an observation sparsity factor $r_{2}$ that measures how much of the surviving signal is captured. Both are individually small, and their product is bounded well below unity for any practical configuration. The posterior is therefore structurally prior-dominated regardless of the number of indirect observations of the same type and configuration—not as an artifact of any method, discretization, or specific PDE, but as a structural property of the information pathway that is computable from the converged Jacobians and the observation geometry alone.

The primary structural determinant of this attenuation is not the PDE class but the coupling mechanism through which ϕ(u) enters the residual. Gradient coupling (diffusion coefficients) yields $r_{1}$ an order of magnitude smaller than pointwise coupling (source terms), making the topology of the prior non-negotiable for diffusion-coefficient identification and producing topology penalties of 5–79× when topology is removed. Pointwise-coupled problems are more forgiving but still benefit substantially from a topological prior.

The framework includes three concrete tools for this analysis: a sparse variational Gaussian process with a fixed analytical mean function that makes the prior explicit and checkable; a closed-form Laplace posterior delivered through the implicit function theorem at no additional cost; and an OED framework that uses direct constitutive measurements to bypass the Jacobian. A small number of well-placed direct measurements—of the order of the number of GP inducing points—collapses the posterior where arbitrarily many indirect observations of the same type cannot. Two further structural diagnostics emerge from the campaign: the spectral concentration $\sigma_{1}^{2}/\Sigma$ refines the Frobenius $r_{1}$ for diffusion-coefficient identification by exposing a symmetry-matching mechanism between the constitutive function and the field’s initial condition, and the Jacobian amplification signature $r_{1}>1$ identifies wrong-local-minimum convergence in elliptic problems where the credible interval would otherwise give no warning.

## Supplementary Material

pgag281_Supplementary_Data

## Data Availability

The code supporting this study will be publicly available at DOI: 10.5061/dryad.pg4f4qs5c.
