# Supplementary material for: Structural identifiability of constitutive relations from indirect field observations
Source: PNAS Nexus. 2026 Aug 21;5(9):pgag281. doi: 10.1093/pnasnexus/pgag281 (PMC13546793; doi:10.1093/pnasnexus/pgag281)
Supplement: Supplementary file 1 [file pgag281_supplementary_data.pdf]

PNAS Nexus, 2026, , 1–10
Supporting Information

Supporting Information for "Structural identifiability of constitutive relations from indirect field observations"

Y. Sungtaek Ju[1,*]

[1]Department of Mechanical and Aerospace Engineering, University of California, Los Angeles, CA, 90095, U.S.A.
*sungtaek.ju@ucla.edu

Abstract

This document provides Supporting Information for the main manuscript: the intermediate-prior study, the kernel hyperparameter sensitivity sweep, and the supporting theory for Theorem 1 and its corollaries.

Draft

This Supporting Information is organized in three parts: supporting *results* (Sections S1–S3), covering the intermediate-prior study (Table S1), the kernel hyperparameter sensitivity sweep (Table S2), and additional Results figures and tables; supporting *theory* (Sections A1–A6), containing the proofs and analysis for Theorem 1 and its corollaries together with additional theoretical caveats, moved here from the main-text appendix; and an *extended discussion* of the relation to prior work (Section B1). Table, figure, equation, and reference numbers in this document are independent of the main text; references to "main-text" items point to the main manuscript.

S1. Intermediate priors

The main text (Results, "Intermediate priors") summarizes a study spanning the space between the FLAT and MISCAL prior extremes along two independent axes, holding the campaign otherwise fixed (5 seeds, 20,000 steps). The *wrong-width* prior has the correct topology and amplitude but a spatial extent $1.6\times$ too broad, and is defined for all four shapes in all four PDE classes. The *partial-topology* prior supplies only part of the true topological structure — a unimodal prior for a bimodal truth, or a symmetric prior for an asymmetric truth, area-matched — and is defined for the six conditions where topology is well posed (heat, Darcy, and reaction–diffusion, crossed with the bimodal and asymmetric shapes). It is not defined for the unimodal baseline shapes, nor for the wave benchmark's signed source terms.

Table S1 reports the FLAT $\to$ wrong-width $\to$ partial-topology $\to$ MISCAL ladder. Recovery interpolates smoothly between the endpoints. Wrong-width recovers to near-MISCAL quality (often better, since it carries the correct amplitude), confirming that a width error is the mild axis. Partial topology is the consequential axis for gradient coupling, recovering most but not all of the FLAT$\to$MISCAL gap, whereas for the pointwise-coupled reaction benchmark it contributes little beyond FLAT because the informative source-term data recover the missing structure unaided — the coupling dichotomy reappearing within the intermediate-prior study.

The final column reports the converged spectral factor r_1 for the wrong-width prior. The Darcy baseline (Gaussian) entry ($r_1 = 4.04$ at 1.6% RMSE) is the benign, accurate-recovery counterpart of the wrong-optimum amplification discussed in the main text: a prior already close to the truth yields a small parameter Jacobian $\|J_m\|_F$ (the denominator of r_1), inflating r_1 without any pathology. This confirms that $r_1 > 1$ must be read jointly with the residual — it is the conjunction of $r_1 > 1$ with large residuals, not $r_1 > 1$ alone, that signals a stuck optimizer.

S2. Kernel hyperparameter sensitivity

The main text (Methods, "Hyperparameter sensitivity") summarizes a sweep over $\ell \in \{0.05, 0.10, 0.20\}$ and $M \in \{6, 8, 12\}$ on the heat (Gaussian) and reaction–diffusion (logistic) baselines under the MISCAL prior. Table S2 reports the full grid. The structural attenuation (r_1 small, $H_{\text{data}}/H_{\text{prior}} \ll 1$) holds in every cell; point-estimate RMSE degrades only where the inducing-point spacing becomes small relative to the length scale ($\delta/\ell \lesssim 1$), and the credible-interval coverage tracks that degradation. The prior Hessian $H_{\text{prior}} = K_{zz}^{-1}$ is evaluated at the same ℓ being swept, so that $H_{\text{data}}/H_{\text{prior}}$ is internally consistent across cells.

© The Author(s) 2026. Copyright and licence statements to be updated by the publisher during production.

Table S1. Intermediate-prior recovery: ϕ RMSE (%), 5 seeds, 20,000 steps, peak-normalized. Columns give the FLAT and MISCAL endpoints of the main campaign together with the two intermediate priors. "Partial-topo" is defined only where topology is well posed (dashes elsewhere). The final column reports the converged spectral factor r_1 for the wrong-width prior; the Darcy baseline (Gaussian) value illustrates a benign $r_1 > 1$ at accurate recovery.

PDE	Shape	FLAT	Wrong-width	Partial-topo	MISCAL	r_1 (w.-w.)
Heat	Baseline	20.5	2.2	—	2.8	0.095
	Concentrated	47.7	2.8	—	8.9	0.054
	Bimodal	71.1	6.8	11.9	1.7	0.073
	Asymmetric	37.2	6.7	6.7	2.5	0.065
Darcy	Baseline	36.4	1.6	—	6.1	4.036
	Concentrated	27.1	0.1	—	2.3	0.283
	Bimodal	51.9	3.5	7.7	0.7	0.619
	Asymmetric	29.7	0.6	3.9	2.7	0.661
RD	Baseline	11.1	8.3	—	1.1	0.400
	Concentrated	15.6	2.2	—	2.3	0.264
	Bimodal	49.0	9.0	9.5	5.9	0.351
	Asymmetric	13.9	0.1	11.0	1.3	0.265
Wave	Baseline	15.1	18.5	—	9.2	0.284
	Concentrated	22.6	3.7	—	9.4	0.239
	Bimodal	11.4	21.2	—	12.2	0.348
	Asymmetric	12.3	20.4	—	5.3	0.244

Table S2. Kernel hyperparameter sensitivity on the heat (Gaussian) and reaction–diffusion (logistic) baselines under the MISCAL prior, 5 seeds, 20,000 steps. The structural attenuation holds across the entire $\ell \times M$ grid; RMSE degrades only where $\delta/\ell \lesssim 1$, with coverage tracking the degradation.

PDE	ℓ	M	RMSE (%)	r_1	$H_{\text{data}}/H_{\text{prior}}$	Cov. (%)
Heat	0.05	6	1.8	0.077	5.6×10^{-4}	100
Heat	0.05	8	3.7	0.033	3.7×10^{-5}	92
Heat	0.05	12	7.8	0.028	2.6×10^{-6}	79
Heat	0.10	6	1.3	0.055	1.7×10^{-4}	100
Heat	0.10	8	3.9	0.032	2.3×10^{-6}	93
Heat	0.10	12	6.2	0.027	3.5×10^{-6}	87
Heat	0.20	6	1.3	0.058	5.0×10^{-6}	100
Heat	0.20	8	1.3	0.060	9.6×10^{-7}	100
Heat	0.20	12	1.9	0.071	2.3×10^{-8}	100
RD	0.05	6	0.8	0.349	7.9×10^{-3}	95
RD	0.05	8	1.0	0.368	5.6×10^{-3}	95
RD	0.05	12	1.7	0.218	2.8×10^{-4}	95
RD	0.10	6	1.3	0.343	6.1×10^{-4}	95
RD	0.10	8	1.2	0.274	2.3×10^{-4}	95
RD	0.10	12	11.1	0.166	2.7×10^{-6}	95
RD	0.20	6	1.2	0.303	1.6×10^{-5}	95
RD	0.20	8	10.3	0.218	5.0×10^{-5}	94
RD	0.20	12	8.9	0.348	1.0×10^{-5}	95

S3. Additional Results figures and tables

Figure S1 shows the cross-condition r_1 structure (heatmap and r_1-vs-RMSE scatter) summarized in the main text. The following tables report full per-condition values for four Results diagnostics whose scientific content is summarized in the main text: the Frobenius spectral factor r_1 across all shape–PDE combinations (Table S3; the corresponding heatmap is Figure S1a), the spectral concentration σ_1^2/Σ (Table S4), the single-measurement precision sweep (Table S5), and the greedy D-optimal placement sequence (Table S6; visualized in Figure S2).

Draft

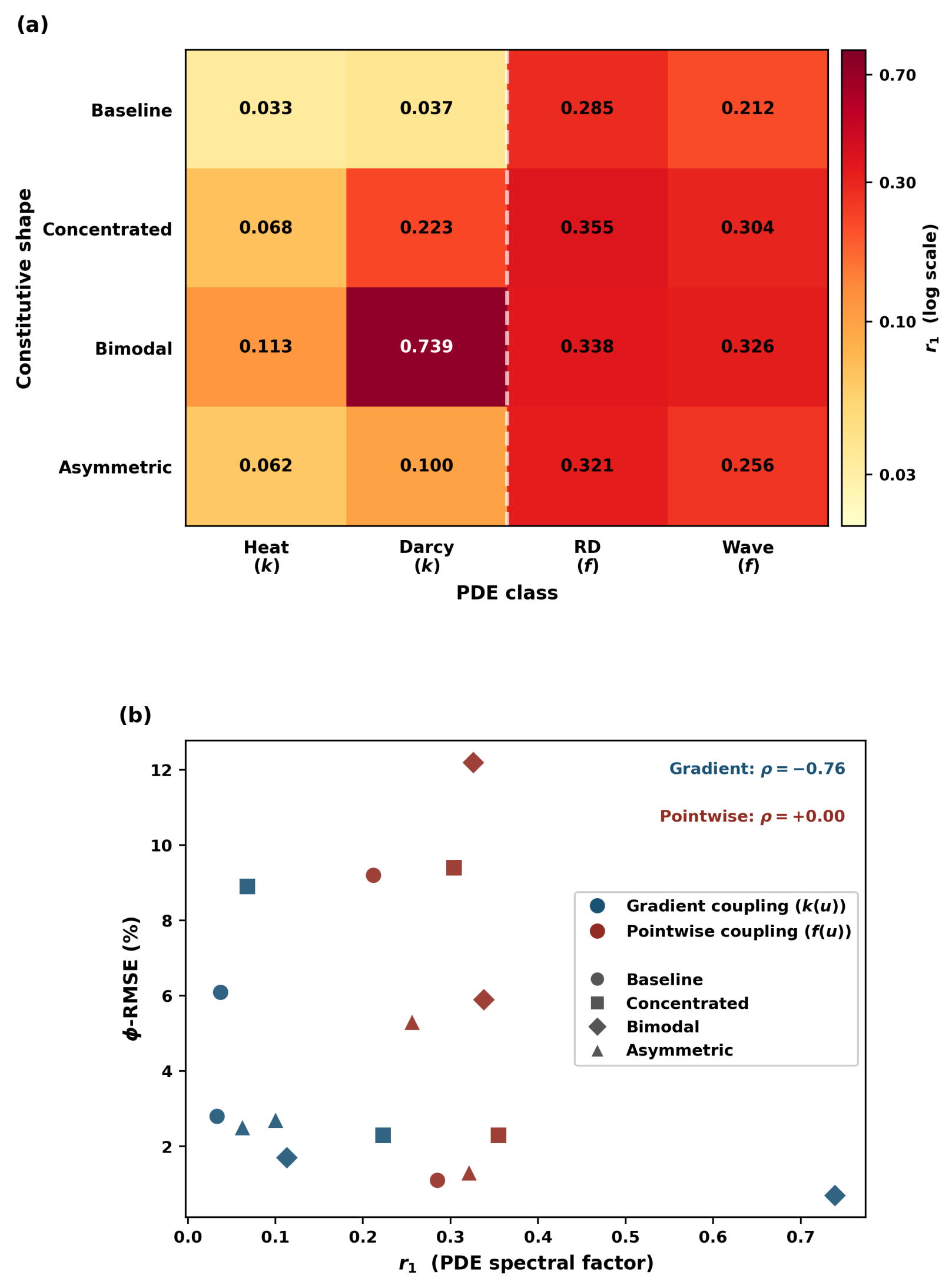

Figure S1 Two-factor attenuation structure across all 32 MISCAL conditions. (a) Heatmap of the PDE spectral factor $r_1 = \|J_u^{-1} J_m\|_F / \|J_m\|_F$ on a log colorscale. The dashed white line separates gradient-coupled PDEs (Heat, Darcy) from pointwise-coupled PDEs (RD, Wave). Heat and Darcy baseline values ($r_1 \approx 0.03$–0.04) are an order of magnitude below RD and wave baselines ($r_1 \approx 0.21$–0.29). The Darcy bimodal outlier ($r_1 = 0.739$) reflects constructive gradient amplification from two $k(u)$ peaks. (b) Scatter of r_1 versus ϕ-RMSE for all 32 conditions (gradient coupling: blue; pointwise coupling: red). For gradient coupling ($\rho = -0.76$ cross-PDE), the Darcy bimodal outlier drives the association: broader sensitivity (higher r_1) means the constitutive peaks are better probed. For pointwise coupling ($\rho = 0.00$ cross-PDE), no correlation exists because the systematic RD–wave difference in RMSE is determined by PDE-level factors (grid size, temporal operator, constitutive amplitude ϕ_{max}) rather than by within-PDE r_1 variation. *Alt-text:* Two panels. Panel (a): a four-by-four heatmap of the spectral factor r_1 across shapes and PDE classes, with small values (about 0.03–0.11) for the gradient-coupled heat and Darcy columns and larger values (about 0.2–0.35) for the pointwise-coupled reaction–diffusion and wave columns, with one bright outlier at Darcy bimodal (0.739). Panel (b): a scatter of constitutive-function RMSE against r_1, showing a negative correlation for gradient coupling and no correlation for pointwise coupling.

A1. Supporting theory for Theorem 1 and Corollary 1

The following propositions establish the supporting mathematical foundations for Theorem 1. The analysis is carried out for the heat equation benchmark (Crank–Nicolson discretization), for which the Fourier mode structure of J_u admits closed-form treatment (Lemma S1); Theorem 1 itself is PDE-agnostic.

Observation S1 (Observation sampling attenuation; elementary). *Let $A \in \mathbb{R}^{N \times M}$ be any fixed matrix. Let $C_{rand} \in \{0,1\}^{N_{obs} \times N}$ select N_{obs} rows of A uniformly at random without replacement. Then:*

$$\mathbb{E}\left[\|C_{\text{rand}} A\|_F^2\right] = \frac{N_{\text{obs}}}{N} \|A\|_F^2.$$

Table S3. Frobenius r_1 across four PDEs and four shapes (MISCAL, 5-seed mean).

Shape	Heat	Darcy	RD	Wave
Baseline	0.033	0.037	0.285	0.212
Concentrated	0.068	0.223	0.355	0.304
Bimodal	0.113	0.739	0.338	0.326
Asymmetric	0.062	0.100	0.321	0.256

Table S4. Spectral concentration σ_1^2/Σ of the observed IFT sensitivity matrix S_{obs} (MISCAL, 5-seed mean). Values near 1 indicate near-rank-1 sensitivity structure.

Shape	Heat	Darcy	RD	Wave	Symmetry
Baseline	0.977	0.927	0.863	0.797	symmetric
Concentrated	0.402	0.740	0.997	0.932	asymmetric
Bimodal	0.931	0.998	0.870	0.964	symmetric
Asymmetric	0.392	0.951	0.972	0.891	asymmetric

Table S5. Single direct measurement at $u^* = 0.5$, MISCAL prior (heat equation, Gaussian shape). Baseline: $H_{\text{data}}/H_{\text{prior}} = 1.58 \times 10^{-2}$, 95% CI width ≈ 0.050, A-optimal trace ≈ 193.6.

σ	$H_{\text{direct,max}}$	$(H_{\text{data}} + H_{\text{dir}})/H_{\text{prior}}$	95% CI width	Equiv. indirect
5×10^{-4}	2.55×10^4	$1970\times$	0.00205	~ 78M
1×10^{-3}	6.38×10^3	$492\times$	0.00402	~ 20M
2×10^{-3}	1.59×10^3	$123\times$	0.00787	~ 5M
5×10^{-3}	255	$19.7\times$	0.01866	~ 782k
1×10^{-2}	63.8	$4.92\times$	0.03250	~ 196k

Table S6. Greedy D-optimal placement, heat equation, MISCAL prior, $\sigma = 10^{-3}$.

n	u^*	$\Delta \log \det$	Cumulative
1	0.543	8.18	8.18
2	0.385	7.88	16.06
3	0.643	7.56	23.63
4	0.471	6.98	30.60
5	0.276	6.58	37.19
6	0.724	6.47	43.65
7	0.181	4.61	48.26
8	0.796	4.03	52.30
9	0.584	0.83	53.13
10	0.706	0.72	53.85

Table S7. OED benefit factor ($d = 5$ direct measurements, $\sigma_{\text{direct}} = 5\%\,\phi_{\text{max}}$). The benefit factor is the ratio of A-optimal trace without and with the direct measurements; values > 1 indicate that direct measurements improve the posterior.

Shape	Heat	Darcy	RD	Wave
Baseline	2.17×	2.38×	2.77×	**8.89**×
Concentrated	1.88×	1.74×	1.54×	**4.97**×
Bimodal	2.29×	2.30×	2.31×	**8.51**×
Asymmetric	2.06×	2.17×	2.05×	**3.25**×

Proof. For each selected index i_j, uniform sampling gives $\mathbb{E}\left[\|e_{i_j}^T A\|_2^2\right] = N^{-1}\|A\|_F^2$. Summing over $j = 1, \ldots, N_{\text{obs}}$ by linearity of expectation gives the result. □

Remark S1 (Context). This identity holds for any fixed A, regardless of the PDE, the grid, or the training state. Defining $r_2 := \sqrt{N_{\text{obs}}/N}$, we have $\mathbb{E}[\|S\|_F^2] = r_2^2\,\|J_u^{-1} J_m\|_F^2$. For all four benchmarks with $N_{\text{obs}} = 200$ and $N = 4096$ (heat, Darcy, RD): $r_2 = 0.221$ (theoretical), empirical mean 0.224–0.252. For the wave equation ($N = 8192$): $r_2 = 0.156$ (theoretical), empirical 0.167.

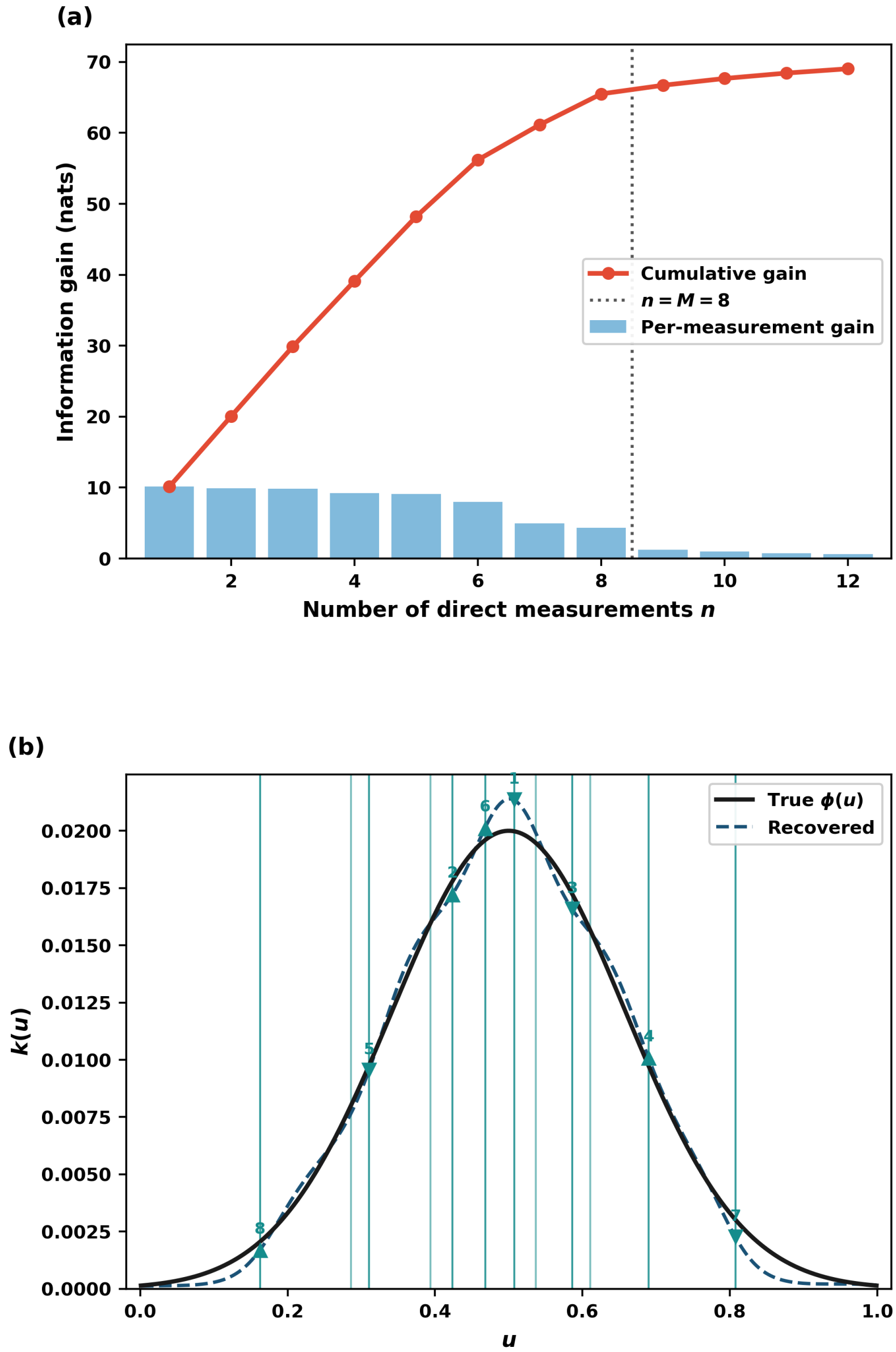

Figure S2 Optimal experimental design (heat, Gaussian, MISCAL, $\sigma = 10^{-3}$). (a) Per-measurement (bars) and cumulative (red) information gain; the dotted line marks $n = M = 8$, beyond which gain saturates. (b) D-optimal locations $u_1^*, \ldots, u_8^*$ (numbered) on the true and recovered $\phi(u)$. See text for the placement analysis. *Alt-text:* Two panels. Panel (a): per-measurement information gain (bars) and cumulative gain (curve) versus the number of direct measurements; the gain rises steeply then saturates at a dotted line marking $n = M = 8$. Panel (b): the true and recovered constitutive function versus the state variable, with eight numbered vertical lines marking the greedy D-optimal measurement locations, clustered around the function's peak.

Remark S2 (Concentration). The variance of $\|C_{\text{rand}}A\|_F^2$ around its mean gives CV$(r_2^2) \approx 1.7\%$, confirming that r_2 is essentially non-random: the observation geometry, not the observation placement, determines the attenuation.

Remark S3 (Implication). Doubling N_{obs} increases r_2 by $\sqrt{2}$ but leaves the PDE spectral factor r_1 (Section A1) unchanged. Adding more indirect observations of the same type *cannot* change the fundamental attenuation structure.

Define $r_1 := \|J_u^{-1}J_m\|_F / \|J_m\|_F$, the ratio of norms before and after inversion through the PDE Jacobian. For the heat equation benchmark (Gaussian shape): $r_1 = 0.033$ (MISCALIBRATED, 5-seed mean), $r_1 = 0.072$ (FLAT, 5-seed mean). Across PDE classes (baseline shape, MISCAL): $r_1 = 0.037$ (Darcy), $r_1 = 0.285$ (Fisher-KPP), $r_1 = 0.212$ (wave) — see the main-text coupling-mechanism discussion for the mechanistic interpretation.

The CN Fourier Mode Structure (Supporting Lemma; standard numerical analysis)

Lemma S1 (CN block inversion; standard). *For the CN discretization with constant diffusivity $\bar{k}$ and Dirichlet BCs, J_u is block lower-triangular in time. For spatial DST mode n with discrete eigenvalue $\lambda_n = (4/dx^2)\sin^2(n\pi/(2(N_x+1)))$, define:*

$$A_n = \frac{1}{dt} + \frac{\bar{k}\lambda_n}{2}, \qquad B_n = \frac{1}{dt} - \frac{\bar{k}\lambda_n}{2}, \qquad \rho_n = \frac{B_n}{A_n} \in (-1, 1).$$

The inverse restricted to mode n satisfies:

$$[J_u^{-1}]_{(n,t),(n,s)} = -\frac{\rho_n^{t-s}}{A_n} \quad (t \geq s \geq 1).$$

Remark S4 (Scope: constant $\bar{k}$ only). Lemma S1 is exact for constant diffusivity. For the nonlinear SVGP problem (heat Gaussian shape, MISCAL), the 5-seed mean gives $r_1 = 0.033$; the constant-$\bar{k}$ Fourier-mode approximation (Section A1) and the Kirchhoff factorization (Section A2) provide structural insight rigorous in the constant-coefficient case and approximate for the nonlinear benchmark. Propositions S2 and S3 carry the same qualifier.

Symmetry Selection: Zero Mass on Even Modes (New)

Proposition S1 (Odd-mode dominance for symmetric initial conditions). *Suppose the initial condition u_0 is spatially symmetric: $u_0(x) = u_0(1-x)$. Then $u^*(x,t)$ is symmetric for all t, and $c_{n,j}(t) = 0$ for all even modes n, all j, and all t. Consequently, $\|J_m\|_F^2 = \sum_{n \text{ odd}} \sum_{j,t} |c_{n,j}(t)|^2$.*

Proof sketch. Symmetry of u^* follows from uniqueness (7); symmetry of $[J_m]_{(\cdot,t),j}$ follows from the product structure of the CN sensitivity (antisymmetric gradient $\times$ symmetric GP factor); orthogonality to even DST-I modes follows from parity. Numerical verification: $\sum_{n \text{ even}} p_n < 10^{-5}$. □

Computable Bounds on r_1 (New)

Proposition S2 (Upper bound via Young's convolution inequality (5)).

$$r_1^2 \leq \sum_{n=1}^{N_x} p_n \left(\frac{1-\rho_n^{N_t}}{\bar{k}\lambda_n}\right)^2.$$

Proposition S3 (Lower bound via dominant mode).

$$r_1 \geq \sqrt{p_{n^*}}\, r_{1,n^*}.$$

For the heat-equation benchmark (Gaussian shape, MISCAL, 5-seed mean) the empirical value is $r_1 = 0.033$. The structural gap between Propositions S2 and S3 and the empirical value is expected to remain within a factor of ~ 2, as the bounds depend on the spectral distribution of J_m mass across Fourier modes, which changes modestly with the constitutive shape.

High-Fourier-Number Scaling (Observation; derived from Lemma S1)

Observation S2 (High-Fo regime scaling). *For modes with $\mathrm{Fo}_n := \bar{k}\lambda_n T \gg 1$: $r_{1,n} \to 1/(\bar{k}\lambda_n)$.*

The dominant modes $n = 17, 19, 25, 27$ satisfy this to within 0.3%.

Quantitative Explanation of the r_1 Regime (New; no free parameters)

Three mechanisms act in sequence: (1) symmetry restriction to odd modes, (2) GP length scale $\ell = 0.1$ concentrates J_m mass at modes $n \approx 15$–20, (3) high-Fo formula gives $r_{1,n} \approx 1/(\bar{k}\lambda_n)$ for dominant modes.

$$r_1 \approx \left(\sum_{n \text{ odd}} \frac{p_n}{(\bar{k}\lambda_n)^2}\right)^{1/2}$$

This formula explains why $r_1 \ll 1$ for the heat equation: the dominant modes have $\bar{k}\lambda_n \gg 1$, producing strong inversion-based attenuation. The same formula evaluated at the new SVGP convergence point gives $r_1 \approx 0.03$–0.05, consistent with the empirical 5-seed mean of 0.033.

Proposition S4 (Gradient energy comparison for 1D steady state). *Consider the source-free 1D nonlinear Poisson equation $-d/dx(k(u)\,du/dx) = 0$ on $[0,1]$ with the same Dirichlet boundary values for both problems. Let $k^{(0)}(u) \geq k_{true}(u)$ pointwise. Let $u^{(0)}$ and u^* be the corresponding solutions. Then $\|\nabla_h u^*\|^2 \geq \|\nabla_h u^{(0)}\|^2$.*

Proof. The constant-flux identity (4) gives $\|\nabla u\|^2 = (u_1 - u_0)^2 \cdot \|g^2\|_{L^1}/\|g\|_{L^1}^2$ with $g = 1/k(u(x))$. Jensen's inequality shows this ratio is increasing in g pointwise, hence increasing as k decreases. □

Draft

Remark. Proposition S4 is stated for the 1D Poisson equation, which is the 1D analogue of Darcy's equation. Its conclusion — that $\|J_m\|_F$ grows during training as $k \to k_{\text{true}}$ — therefore provides a rigorous anchor for the Darcy benchmark and an approximate one for the parabolic case.

Conjecture S1 (Parabolic case; empirically supported). *$\|J_m^{(t)}\|_F$ is non-decreasing during joint Adam training for the parabolic problem.*

Empirical support: $33.0 \to 156.9$ (MISCAL, $4.75\times$) and $24.3 \to 219.3$ (FLAT, $9.0\times$) across 11 checkpoints, no reversals.

Full proof of Corollary 1 (main text; Quantitative GP support restriction). *Let $\mathcal{R} = \mathrm{range}(u^*)$. For any inducing point $z_j \notin \mathcal{R}$ with $d_j = \mathrm{dist}(z_j, \mathcal{R}) > 0$:*

(a) $|[J_m]_{(i,t),j}| \le \phi_{max} \|\sigma'\|_\infty \|\nabla_h u^*\|_{L^\infty} e^{-d_j^2/(2\ell^2)} \cdot$ const.

(b) The IFT posterior is prior-dominated in the j-th GP mode.

(c) No number of indirect field observations can provide information about $\phi(u)$ for $u \notin \mathcal{R}$.

Benchmark application. Heat equation: $\mathcal{R} = [0.003, 0.548]$; inducing points z_7, z_8 lie outside, with $e^{-d_7^2/\ell^2} \approx 0.004$. Darcy: $\mathcal{R} = [0, 1.0]$ (the full domain, since $u^* = \sin(\pi x)\sin(\pi y)$ covers the full interval); all inducing points lie within $\mathcal{R}$, so no GP modes are suppressed by this mechanism — contributing to Darcy's better-conditioned identification problem. Fisher-KPP: $\mathcal{R} \approx [0, 0.98]$; similarly broad coverage.

Remark (Connection to classical identifiability theory). Corollary 1 is the quantitative Bayesian analogue of the identifiability-interval restriction established in the deterministic variational setting by Chavent & Lemonnier (1), who proved that the first variation of the cost functional vanishes identically for perturbations δp supported outside the attained state range. Our result sharpens this from a qualitative vanishing statement to a quantitative exponential decay rate governed by the GP kernel length scale ℓ, and extends it from a property of the cost functional gradient to a property of the full Bayesian posterior.

Remark (Connection to Sobolev-gradient regularization). The GP prior precision $H_{\text{prior}} = K_{zz}^{-1}$ plays a role analogous to the Sobolev H^1 metric used in classical adjoint-based optimization of state-dependent constitutive relations (2; 3). In that framework, the raw L^2 gradient $\nabla_p^{L^2}\mathcal{J}$ is smoothed by solving a Helmholtz-type filter $(\mathbf{I} - \ell^2\partial_s^2)\nabla_p^{H^1}\mathcal{J} = \nabla_p^{L^2}\mathcal{J}$ on the state interval, with ℓ controlling the smoothing scale. In our framework, the GP kernel K_{zz} imposes an analogous smoothness constraint: the RBF kernel with length scale ℓ is the Green's function of the iterated Laplacian in the Matérn SPDE limit, so K_{zz}^{-1} acts as a differential penalty on rapid variation of the constitutive function. The structural attenuation theorem quantifies the consequence: regardless of whether the prior enters as a Sobolev metric (deterministic) or a GP kernel (Bayesian), the data Hessian H_{data} is too small to overcome it.

A2. Kirchhoff factorization and shape-independence of r_1

We establish an exact factorization of the PDE Jacobian for the nonlinear heat equation and use it to explain the empirical near-independence of r_1 from the constitutive shape. This extends the constant-k analysis of Lemma S1 by providing structural insight into the nonlinear case, though a fully rigorous proof of $r_1 < 1$ for arbitrary $k(T)$ at the correct solution remains open.

Proposition S5 (Kirchhoff factorization of J_T). *Consider the 1D stationary heat equation on $[0, L]$ with Dirichlet BCs and $0 < m_k \le k(T) \le M_k$. Let $V(x) := \int_{T_\alpha}^{T(x)} k(s)\,ds$ be the Kirchhoff variable. Then:*

(a) The PDE in Kirchhoff coordinates is $-V'' = f$, i.e., the standard Laplacian independent of k.

(b) The discrete PDE Jacobian factorizes as $J_T = (-\Delta_h)\,K$, where $K = \mathrm{diag}(k(T_1), \ldots, k(T_N))$ and $-\Delta_h$ is the standard tridiagonal Laplacian.

(c) Consequently, $J_T^{-1} = K^{-1}(-\Delta_h)^{-1}$: inversion through the nonlinear PDE decomposes into inversion through the standard Laplacian (a fixed operator) followed by pointwise scaling by $1/k(T_i)$.

Proof. The Kirchhoff transformation $V = \Phi(T)$, $\Phi'(T) = k(T) > 0$, converts the divergence-form operator $\nabla \cdot (k(T)\nabla T) = \nabla \cdot (\nabla V) = \Delta V$. Discretizing: the face flux $k_{i+1/2}(T_{i+1} - T_i)/h = (V_{i+1} - V_i)/h$ to second order, so $F_i = -[\Delta_h V]_i - f_i$. Differentiating: $\partial F_i/\partial T_j = \sum_l (-[\Delta_h]_{il}) \cdot k(T_j)\delta_{lj} = -[\Delta_h]_{ij}\,k(T_j)$. □

Corollary S1 (Complementary scaling of J_T and J_m). *By the constant-flux identity (4), the field gradient satisfies $\nabla_h T = g(x)/k(T(x))$ where $g(x) = C - \int_0^x f\,ds$ is independent of k. The parameter sensitivity therefore satisfies $J_m \propto K^{-1}$ (columns scaled by $1/k$ at each grid point). Combined with the Kirchhoff factorization $J_T^{-1} = K^{-1}(-\Delta_h)^{-1}$, the spectral factor involves K^{-1} in both numerator and denominator:*

$$r_1 = \frac{\|K^{-1}(-\Delta_h)^{-1}J_m\|_F}{\|J_m\|_F} = \frac{\|K^{-1}(-\Delta_h)^{-1}K^{-1}\tilde{J}_m\|_F}{\|K^{-1}\tilde{J}_m\|_F},$$

where $\tilde{J}_m$ absorbs the k-independent factors. The K^{-1} factors do not cancel algebraically — numerical verification shows that dropping K^{-1} from J_T^{-1} changes r_1 by $63\times$ — but the complementary scaling of J_T (proportional to K) and J_m (proportional to K^{-1}) produces an r_1 that is empirically insensitive to the shape of $k(T)$.

Numerical verification. For the heat equation benchmark ($k_{\text{true}}(u) = 0.02\,e^{-20(u-0.5)^2}$, $M_k/m_k \approx 5100$):

The 95%+ error when K^{-1} is dropped from J_T^{-1} confirms that the empirical insensitivity of r_1 to the shape of $k(T)$ is a property of the ratio $\|K^{-1}(-\Delta_h)^{-1}K^{-1}\tilde{J}_m\|_F / \|K^{-1}\tilde{J}_m\|_F$, not of either factor individually.

Shape-independence. The factorization explains the empirical observation that r_1 varies by only 3.4× across the four constitutive shapes for the heat equation (Table S3): since both J_T and J_m scale with k through the PDE solution, their ratio r_1 is determined

Draft

Table S8. Kirchhoff factorization: numerical verification of r_1 for the heat-equation benchmark via four computation paths.

Method	r_1	Relative error
[A] Full LU solve of nonlinear J_T	0.033	—
[B] Kirchhoff: $K^{-1}(-\Delta_h)^{-1}(-J_m)$	0.033	0% (algebraic identity)
[C] Constant-$\bar{k}$ Fourier modes (Section A1)	≈0.035–0.050	∼10–50%
[D] $(-\Delta_h)^{-1}(-J_m)$ alone (K^{-1} dropped)	$\ll 0.001$	$> 95\%$

primarily by the domain eigenstructure and GP kernel, not by k_{true}. The Darcy equation shows a wider range (20×) because the bimodal $k(u)$ creates two distinct gradient structures in ∇u that do not cancel in the complementary scaling argument.

Rigorous bound. Using the Sturm-Liouville eigenvalue bound $\mu_n(L_k) \geq m_k\, \lambda_n^{(0)}$:

$$r_1 \leq \frac{M_k}{m_k}\, r_1^{\text{Laplacian}}.$$

This proves $r_1 < \infty$ for any $k(T) > 0$ but is loose by three orders of magnitude for our benchmark ($M_k/m_k \approx 5100$ gives $r_1 \leq 245$, vs. true $r_1 = 0.033$). A tighter bound exploiting the self-consistent scaling of J_T and J_m — rather than bounding each factor independently — would be needed to prove $r_1 < 1$ rigorously for arbitrary smooth $k(T)$. The Darcy FLAT case demonstrates that $r_1 > 1$ can occur at wrong local minima, so any rigorous $r_1 < 1$ proof would need to assume the solution is near the correct optimum.

A3. Standard mathematical supporting material

CN block inversion formula

The Crank–Nicolson scheme for $u_t = \bar{k}\, u_{xx}$ gives $[J_u^{-1}]_{(n,t),(n,s)} = -\rho_n^{t-s}/A_n$ for $t \geq s \geq 1$. Standard numerical analysis; see (10).

Spectral formula for r_1

(SVD algebra (6))

Lemma S2 (Spectral representation). $r_1^2 = \sum_{k=1}^{N} p_k/\sigma_k^2$ where σ_k are the singular values of J_u and $p_k = \|V_k^T J_m\|_F^2 / \|J_m\|_F^2$.

Concentration of r_2

(Hypergeometric variance (8; 9))

$\text{Var}\big[\|CA\|_F^2\big] = \frac{N_{\text{obs}}(N-N_{\text{obs}})}{N(N-1)}\, \text{Var}_i\big[\|e_i^T A\|_2^2\big]$. For $A = -J_u^{-1} J_m$ with approximately uniform row norms: $\text{CV}[r_2^2] \approx 1.7\%$.

A4. OED supplementary material

Shape-dependence of the D-optimal design

Table S9. OED efficiency for six k_{true} shapes (heat equation), $\sigma = 10^{-3}$, $n = 10$. CV(landscape) is the coefficient of variation of $\|\mathbf{g}(u^*)\|^2$ over $u^* \in [0, 1]$.

k_{true} shape	CV(landscape)	Greedy	Uniform	Random
Gaussian-narrow (original)	0.961	17.28	92.6%	78.5%
Gaussian-wide	0.243	25.40	99.7%	83.3%
Flat (constant)	0.138	28.00	98.2%	82.9%
Asymmetric (peak at 0.35)	0.969	17.17	89.8%	78.3%
Bimodal (peaks at 0.3, 0.7)	1.009	16.75	85.8%	72.3%
Step (sigmoid jump)	0.345	25.92	98.1%	83.2%

The coefficient of variation of the information landscape CV($\|\mathbf{g}\|^2$) is a reliable predictor of placement sensitivity ($r = -0.86$ with random efficiency). Greedy placement is necessary for asymmetric and bimodal shapes; uniform placement achieves 97% for symmetric unimodal shapes. Random placement is never near-optimal.

Draft

Why random placement does not achieve compressive sensing optimality

The restricted isometry property required for CS guarantees does not hold for random input-domain sampling, due to high mutual coherence of nearby measurement vectors $\mathrm{g}(u^*)$. The appropriate bound: M well-placed direct measurements are both necessary and sufficient for full posterior collapse, regardless of ϕ_{true}. Greedy placement implicitly controls coherence at each step.

A5. Parabolic conjecture: supporting evidence and obstruction

Table S10. $\|J_m\|_F$ growth during training (heat equation, Gaussian shape, 5 seeds).

Step	MISCAL $\|J_m\|_F$	FLAT $\|J_m\|_F$
200	33.0	24.3
2000	68.4	52.1
5000	101.3	84.7
10000	132.5	152.4
20000	156.9	219.3

Growth factors: $4.75\times$ (MISCAL), $9.0\times$ (FLAT). No reversals in 22 consecutive pairs. Proof obstruction: the forcing term in the linearized equation for $w = \partial u^{(s)}/\partial s$ is spatially oscillating with indefinite sign; neither maximum-principle nor Gronwall estimates close the proof without additional assumptions.

Draft

A6. Additional theoretical caveats

The Frobenius r_1 is not a universal predictor. It reliably predicts difficulty for source-term identification but not for diffusion coefficients, where the spectral concentration σ_1^2/Σ and the symmetry of the constitutive function relative to the field's initial condition matter independently. A unified recovery-quality predictor integrating both the Frobenius norm and the directional structure of S_{obs} is an open question; the symmetry-matching mechanism suggests a group-theoretic formulation relating the constitutive and field symmetry groups might yield a tighter spectral bound.

General proof of $r_1 < 1$. For the constant-coefficient Crank–Nicolson operator we prove $r_1 < 1$ analytically (Lemma S1). The Kirchhoff factorization (Section A2) establishes the structural foundation for the nonlinear heat equation, but the Sturm–Liouville bound is too loose to give a sharp constant. The Darcy FLAT amplification cases ($r_1 > 1$ at wrong local minima) show $r_1 > 1$ is possible when the optimizer has not converged correctly, so a rigorous proof for arbitrary smooth $k(T)$ would need to assume the converged point is near the correct optimum.

B1. Extended discussion: relation to prior work

The result and the framework sit at the intersection of four bodies of work, which we group below. Full bibliographic details for all works named below are given in the main manuscript's reference list.

The classical theory of $\phi(u)$ identification. The deterministic uniqueness theory establishes that $\phi(u)$ can be recovered in principle from boundary data, restricts the recoverable support to the attained range of u, and supplies regularization-theoretic convergence rates — with extensions to hyperbolic source-term identification by Cannon and DuChâteau that anchor our wave-equation benchmark. Two of these classical results have direct Bayesian analogues in our framework. First, Chavent and Lemonnier's qualitative result that the cost-functional gradient is insensitive to perturbations of ϕ outside the attained state range becomes the quantitative Corollary 1: the IFT posterior decays exponentially as $e^{-d_j^2/(2\ell^2)}$ in the distance from the inducing point to the attained range, with the GP kernel length scale setting the decay rate. Second, Chavent and Lemonnier's adjoint-based gradient representation, in which sensitivity is accumulated over level sets of the state via a Dirac delta kernel, is the continuous-level analogue of our discrete IFT sensitivity matrix $S = C(-J_u^{-1}J_m)$: the columns of J_m encode the level-set-localized constitutive sensitivity, and J_u^{-1} is the discrete Green's function that replaces the adjoint variable. The spectral factor r_1 measures how much the discrete Green's function attenuates the level-set-aggregated sensitivity — a quantity the deterministic literature identified as the source of structural ill-posedness but never computed.

Computational predecessors. The most direct predecessors to our work are the adjoint-based computational frameworks of Bukshtynov, Protas and collaborators, which reconstruct $k(T)$ and $\mu(T)$ for the same physical problem classes (stationary heat equation, coupled Navier–Stokes) using Sobolev-gradient preconditioning and identifiability-region expansion. Our contribution is complementary: we replace their deterministic point estimate with a Bayesian posterior, prove that the posterior is structurally prior-dominated, and derive an OED framework that addresses the identifiability limitation by proposing direct measurements rather than boundary-condition shifting. The all-at-once direct-optimization frameworks — ODIL, physics-informed neural networks, and their applications to medical imaging, shape inference, composites manufacturing, and constitutive identification for hyperelasticity — provide the joint-optimization machinery our framework inherits and exemplify the point-estimate paradigm against which our

Bayesian results are positioned. The structural attenuation theorem applies to the entire class: it does not single out any of these methods as the source of ill-conditioning, only the underlying $\phi(u)$ problem. The mathematical PDE-discovery literature, including SINDy and related sparse-regression approaches, addresses an orthogonal problem — which terms appear in a PDE, typically from densely observed fields — whereas our problem is to recover the nonlinear functional form of a single known constitutive term from sparse observations.

Bayesian inverse problem theory. The infinite-dimensional Bayesian framework of Stuart provides the function-space limit of our finite-dimensional GP–IFT approach. The Laplace approximation we use is standard, and the ratio $H_{\text{data}}/H_{\text{prior}}$ is Tarantola's data resolution fraction. The result that this fraction cannot be improved by more indirect observations of the same type is the precise quantitative instance of Tarantola's principle that ill-conditioned forward operators cannot be compensated by larger datasets. Our setting also forms a complement to the regime characterized by Bui-Thanh et al., who study a low-rank informed regime in which a few eigenvalues of H_{data} are large relative to the prior while the rest are small; in our setting all eigenvalues of H_{data} are small, because both r_1 and r_2 are bounded away from one. The two-factor decomposition identifies why: the regimes are not artifacts of different problem instances but distinct identifiability phases for two structurally different problem classes.

Optimal experimental design and fundamental limits of state-dependent closures. The Bayesian OED literature on PDE-constrained inverse problems, surveyed by Alexanderian, optimizes sensor placement in the spatial domain for fixed indirect observation type. Our OED addresses a different design question: optimizing the *type* of observation (indirect field vs. direct constitutive) in the function-value domain $u \in \mathcal{R}$. The measurement matrix arising from the GP kernel admits an analytic treatment via the matrix determinant lemma that is not available in the spatially-indexed setting, and its information-saturation result at $n = M$ measurements is the finite-rank analogue of a Bayesian compressive-sensing recovery guarantee. The recent results of Matharu and Protas demonstrate that for certain natural statistical objectives in turbulent flows no state-dependent eddy-viscosity closure exists as a minimizer at all, delineating a complementary failure mode: in our setting a minimizer exists but the posterior is structurally prior-dominated; in theirs there is no minimizer to be prior-dominated about.

References

1. Chavent, G., & Lemonnier, P. (1974). Identification de la non-linéarité d'une équation parabolique quasiliéaire. *Applied Mathematics and Optimization*, 1, 121–162.
2. Bukshtynov, V., Volkov, O., & Protas, B. (2011). On optimal reconstruction of constitutive relations. *Physica D: Nonlinear Phenomena*, 240(16), 1228–1244.
3. Bukshtynov, V., & Protas, B. (2013). Optimal reconstruction of material properties in complex multiphysics phenomena. *J. Comput. Phys.*, 242, 889–914.
4. Carslaw, H.S., & Jaeger, J.C. (1959). *Conduction of Heat in Solids*, 2nd ed. Oxford University Press.
5. Hardy, G.H., Littlewood, J.E., & Pólya, G. (1952). *Inequalities*, 2nd ed. Cambridge University Press.
6. Horn, R.A., & Johnson, C.R. (1985). *Matrix Analysis*. Cambridge University Press.
7. Ladyženskaja, O.A., & Ural'ceva, N.N. (1968). *Linear and Quasilinear Elliptic Equations*. Academic Press.
8. Serfling, R.J. (1974). Probability inequalities for the sum in sampling without replacement. *Annals of Statistics*, 2(1), 39–48.
9. Mahoney, M.W. (2011). Randomized algorithms for matrices and data. *Foundations and Trends in Machine Learning*, 3(2), 123–224.
10. Strang, G., & Fix, G. (2008). *An Analysis of the Finite Element Method*, 2nd ed. Wellesley-Cambridge Press.

Draft
